# Underlying Subwavelength Aperture Architecture Drives the Optical Properties of Microcavity Surface Plasmon Resonance Sensors

**DOI:** 10.3390/s20174906

**Published:** 2020-08-30

**Authors:** Dragos Amarie, Nazanin Mosavian, Elijah L. Waters, Dwayne G. Stupack

**Affiliations:** 1 Department of Physics and Astronomy, Georgia Southern University, Statesboro, GA 30560, USA; ew03271@georgiasouthern.edu; 2 Optical Science and Engineering, University of New Mexico, Albuquerque, NM 87106, USA; nmosavian@unm.edu; 3 Department of Reproductive Medicine, School of Medicine and Moores Cancer Center, University of California San Diego, La Jolla, CA 92093, USA; dstupack@ucsd.edu

**Keywords:** surface plasmon resonance, standing surface plasmon waves, surface plasmon polaritons, optical biosensors, microcavity surface plasmon resonance sensors, MSPRS

## Abstract

Microcavity surface plasmon resonance sensors (MSPRSs) develop out of the classic surface plasmon resonance technologies and aim at producing novel lab-on-a-chip devices. MSPRSs generate a series of spectral resonances sensitive to minute changes in the refractive index. Related sensitivity studies and biosensing applications are published elsewhere. The goal of this work is to test the hypothesis that MSPRS resonances are standing surface plasmon waves excited at the surface of the sensor that decay back into propagating photons. Their optical properties (mean wavelength, peak width, and peak intensity) appear highly dependent on the internal morphology of the sensor and the underlying subwavelength aperture architecture in particular. Numerous optical experiments were designed to investigate trends that confirm this hypothesis. An extensive study of prior works was supportive of our findings and interpretations. A complete understanding of those mechanisms and parameters driving the formations of the MSPRS resonances would allow further improvement in sensor sensitivity, reliability, and manufacturability.

## 1. Introduction

The surface plasmon resonance (SPR) phenomenon has been studied extensively. Since the late 1960s, exponential growth in the number of annual publications has been recorded [1,2]. The history of this field [1,2,3], classic reviews of the surface plasmons (SPs), the surface plasmon polaritons (SPPs), also known as surface plasmon waves (SPWs), the SPR on flat metal films [4,5,6], the main differences between SPs and localized surface plasmons (LSPs) [7,8,9,10,11] as well as their applications were previously discussed [12,13,14,15].

The need for the detection of numerous chemical and biological species in a multitude of scenarios and geometrical configurations has generated a wealth of applications in the field of SP-based biosensor development, a field that is continuously growing in diversity with applications. Aside from key roles in food safety and homeland security, there is a burgeoning field in molecular biology and medical diagnostics [13,14,16]. SP-based biosensors are optimal for the detection of the products from single cells, thus allowing researchers to directly address heterogeneity on the single cell level [17,18,19]. Moreover, recent global events highlight important applications in environmental monitoring [20]. This multitude of SP-biosensor developments come from the fact that the SPP properties are highly dependent on excitation method, as well as the dimensions and the geometries of the metal sustaining the SPs [2].

Of significance for this study is the situation when one dimension of the SP support metal is larger than SPP wavelength but shorter than its propagation length. As such, the SPP can reflect from structure’s edge and interfere with itself, thus producing a standing SPW (SSPW) across the structure. For example, Wanstall et al. excite SSPWs in strongly blazed overhanging zero-order metal gratings [21], whereas Rochon and Lévesque create SSPWs by generating two counter-propagating plasmon waves from two superimposed grating patterns that couple light to SPP at a metal-air interface [22]. Passian et al. delocalized SSPW in a Kretschmann configuration (KC) by interfering two anti-parallel SPPs [23]. Kwak et al. use a near-field probe to induce SSPWs between adjacent 250-nm apertures of a periodic array on 100-nm thick Au and Ni-Au films [24]. Li et al. excite multiple SSPWs on 1-arm (I-shape), 2-arm (L-shape), and 3-arm (Z-shape) Ag nano-rods and correlate the length of the resonator to SSPW wavelengths [25]. To date, these works were most influential in understanding the physics governing microcavity surface plasmon resonance sensors (MSPRSs) [26,27,28].

MSPRS is an attractive sensor for several chief attributes. Firstly, MSPRS has about one micron footprint, thus making it a prime candidate for lab-on-a-chip sensing applications as it integrates with microfluidics devices [28]. Secondly, MSPRS is comparable in size with cells or bacteria. Its nearby location would allow investigations at a single cell or single bacterium level. Thirdly, MSPRS sensing surface is estimated at about 2.5 µm^2^. One single MSPRS is capable of detecting about 35,000 glucose oxidase molecules representing 9.6 fg or 60 zmol of protein [27]. By comparison, this is about a million times less amount of mass than is needed for classic SPR. We have previously demonstrated that selected MSPRSs are about 250 times more sensitive than the Biacore 3000 when benchmarked against the same signal-to-noise ratio [27]. Lastly, the MSPRS works in transmission mode and it does not require goniometric alignment, polarized incident light, precise wavelength of excitation light, and it is independent of the excitation method. When optically investigated, the MSPRS emitted light shows a characteristic pattern dominated by spectral peaks. The internal MSPRS morphology and the underlying subwavelength aperture architecture, in particular, affect their mean wavelength, peak width, and peak intensity.

The focus of this work is to explore the mechanisms driving the formations of MSPRS resonances. A conceptual approach to SPP, SPR, and SSPW is presented in Section 3.1. KC, as a supporting platform for the development of the MSPRS technology, is explored in Section 3.2. The complexities associated with the MSPRS design and the current state of the art in the field are summarized in Section 3.3, Section 3.4 and Section 3.5. MSPRS design principles are detailed in Section 4.1. Its practical implementation and differences between design and experimental outcome are discussed in Section 4.2. The rest of Section 4 discusses multiple levels of experimental evidence and supporting analyses to promote the *hypothesis*: SSPWs were sustained at the surface of the MSPRS and that their coupling back into propagating photons was responsible for the emission of the observed MSPRS resonances.

## 2. Materials and Methods

**Figure 1:**
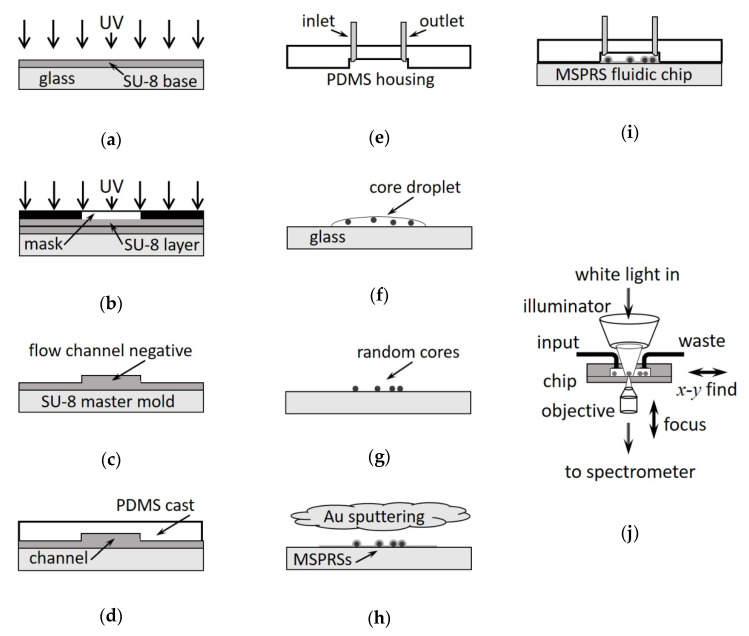
Main steps in microcavity surface plasmon resonance sensor (MSPRS) chip fabrication. (**a**) An adhesion SU-8 base layer was made on a clean glass slide; (**b**) The microfluidic channel pattern was transferred from a transparent mask to the second SU-8 layer; (**c**) The un-exposed SU-8 was removed. The SU-8 master mold shows the flow channel negative features; (**d**) The microfluidic housing was made by casting poly-dimethyl-siloxane (PDMS) over the SU-8 master; (**e**) Fluid inlets and outlets were bored into the PDMS housing and the tubing was attached; (**f**) The MSPRS substrate was made by dispensing a droplet of polystyrene cores on a cover glass; (**g**) This droplet was dried out revealing cores randomly distributed on glass; (**h**) Au was sputtered over both the cores and the glass substrate resulting in MSPRSs; (**i**) An MSPRS chip was made by assembling the PDMS housing from (**e**) and the MSPRS substrate from (**h**); (**j**) Schematic representation of the optical setup.

To be able to investigate MSPRS resonances under changes in the refractive index of the medium in contact with the sensor surface, populations of MSPRSs were encapsulated inside of a microfluidic device. To fabricate a fluidics housing for the sensors (the MSPRS chip), two components are required: a MSPRS substrate and a microfluidics chamber. The MSPRS substrate was made by a transparent cover glass that supports the Au film deposited over a large mono-disperse population of polystyrene cores. To manipulate the fluid flow at the sensor’s surface, we casted a microfluidic chamber in a transparent poly-dimethyl-siloxane (PDMS) elastomer from a SU-8 negative photoresist master mold. These two components were assembled into a MSPRS chip. Individual MSPRSs were interrogated in transmission mode using an inverted microscope. While illuminated with white light from above, the MSPRS emitted light was collected from below and spectrally analyzed. The following protocols are an updated version of previously used protocols [28]. Figure 1a–i show the main steps in MSPRS chip fabrication. Figure 1j shows a schematic of the optical setup.

### 2.1. Microfluidic Mold Fabrication

To fabricate a microfluidics housing, we used PDMS to replicate the channel structure from a master template. The microfluidic structure, a T-junction with two input channels (50 μm × 20 mm) connecting into a common output chamber (1 mm × 20 mm), was drawn in AutoCAD (AutoDesk, Inc., San Rafael, CA, USA) and printed on a high neutral density transparency photomask using a high-resolution laser photo-plotter (40,640 dpi, PhotoPlot Store, Inc., Colorado Springs, CO, USA). The channel structure was then transferred to a SU-8 layer (negative tone photoresist, Kayaku Advanced Materials, Westborough, MA, USA). To make master substrate, we cleaned glass slides (75 × 50 × 1 mm^3^, Corning Inc. Corning, NY, USA) in 300 mL of 1:1:1 solution of NH_4_OH (14.8 M, EMD, MilliporeSigma, Burlington, MA, USA), H_2_O_2_ (36%, Macron Fine Chemicals, Avantor, Radnor, PA, USA), and ultra-pure water (Type 1 Ultra-Pure 18.2 MΩ H_2_O, Barnstead Thermolyne NanoPure, Thermo Fisher Scientific Inc., Waltham, MA, USA) at 75 °C for 1 hr or until the boiling stopped. We rinsed the glass substrates abundantly with ultra-pure water, methanol (99.8%, EMD, MilliporeSigma, Burlington, MA, USA), and then dried them under a stream of nitrogen at 80 psi (Industrial Grade, Airgas, Inc., Radnor, PA, USA). The master was fabricated with two SU-8 layers. The first layer, about 20 μm thick, promoted the adhesion of the channel structure (embedded in the second layer) to the glass slide (Figure 1a). A spin-coater (WS-400B-6NPP, Laurell Technologies, Co., North Wales, PA, USA) was used to apply SU-8 photoresist onto the glass slides by ramping at 106 rpm/s to 1,000 rpm for 30 s. We pre-baked the photoresist on a digital hotplate (PMC 732P, Barnstead Thermolyne Corp., Ramsey, MN, USA) at 65 °C for 1 min, then ramping them to 95 °C at 100 °C/hr and holding for 3 min. We processed both layers identically, except that the first layer was exposed to UV without a photomask. The UV-light (365 nm, 150 mJ/cm^2^; 205S, Optical Associates, Inc., San Jose, CA, USA) from a high-pressure Hg-arc lamp was passed through an additional narrow band pass filter with 360 nm mean wavelength and 45 nm full width at half maximum intensity (Edmund Optics, Inc., Barrington, NJ, USA). The microfluidic pattern was, therefore, transferred from the photomask onto the second SU-8 layer (Figure 1b). We post-baked the exposed photoresist at 65 °C for 1 min, then ramping to 95 °C at 300 °C/hr, and holding for 1 min. The un-polymerized photoresist was removed by soaking in SU-8 Developer (Kayaku Advanced Materials, Westborough, MA, USA) for 10 min, then rinsing the substrate with 2-propanol (Sigma-Aldrich, MilliporeSigma, Burlington, MA, USA) and drying it gently with nitrogen. The SU-8 ridge (Figure 1c, flow channel negative) was 22.7 ± 0.8 μm tall measured (N = 15) across the structure with a stylus profiler (Dektak 6M, Veeco Corp., Plainview, NY, USA).

### 2.2. Microfluidic Chamber Fabrication

Microfluidic structures were cast in PDMS (refractive index n=1.417±0.005) [29] by mixing 1:10 base to curing agent (Sylgard 184, Dow Corning Corp., Midland, MI, USA). A tape barrier was constructed around the master to hold the PDMS mixture poured over the SU-8 pattern (Figure 1d). The PDMS mixture was degassed under low vacuum (about 1 Torr) for an hour (or until all the air bubbles were purged out), then baked at 100 °C for one hour. The PDMS cast was separated from the master while hot to prevent its adhesion to SU-8 mold. Inlet and outlet holes were punched in PDMS with a 16-G titanium-nitride coated needle and the edges were trimmed off (Figure 1e). The PDMS cast and the MSPRS substrate were oxidized under a 300-mTorr air plasma (PDC-32G, Harrick Plasma, Ithaca, NY, USA) for 2 min and 45 s. The MSPRS substrate and the PDMS chamber were brought in contact immediately and allowed to bond overnight under pressure (1 kg of pressure). The flow rate was controlled with programmable syringe pumps (Infusion Pump-200, KD Scientific, Inc., Holliston, MA, USA) connecting the liquid reservoirs to the microfluidic ports with Tygon tubing (1.6 mm OD, VWR International, Radnor, PA, USA).

### 2.3. MSPRS Substrate Fabrication

Borosilicate cover glass substrates (No. 1.5, 50 × 24 mm^2^, refractive index n=1.525±0.002, VWR International, Radnor, PA, USA) [30] were cleaned as presented above. The MSPRS cores were purchased as an aqueous stock solution of polystyrene nanospheres 780±6 nm in diameter with a refractive index of n=1.59±0.01 (Bangs Laboratories, Inc., Fishers, IN, USA) [31]. We sonicated a 10 μL stock solution in 2 mL isopropanol (Chromatography Grade, EMD, MilliporeSigma, Burlington, MA, USA) for 1.5 min to break any clumped particles. A 3-μL sample solution was dispensed on the glass substrate. The droplet spread over the entire substrate and was let dry for 5 to 10 min (Figure 1f). Dried substrates were investigated under a microscope (10× objective) for minimal clusters composition and core distribution (Figure 1g). A desired distribution had cores well-spaced, 10 to 20 μm apart over a 1 mm^2^ field of view. Selected substrates were then coated with 170 nm Au film under a sputtering current of 100 mA (K-675D Dual-Target Turbo Sputter Coater, Quorum Technologies Ltd., Laughton, East Sussex, UK) unless specified otherwise (Figure 1h). A focused ion beam (FIB, Auriga-60, Carl Zeiss Microscopy, LLC., White Plains, NY, USA) operated in CrossBeam mode used gallium ions to etch selected areas to reveal the MSPRS inner structure and thus allowed for dimensional measurements.

### 2.4. Optical Setups and Data Analysis

Two optical setups were used to collect the light emitted by MSPRSs. Spectral data collected on these setups were independent of each other and used for different experiments and analyses, therefore no cross-calibration or additional control experiments were required or performed.

The first setup used an inverted microscope (Diaphot, Nikon, Inc., Melville, NY, USA) with 0.3 numerical aperture (NA) condenser and an air objective (40×, 0.85 NA, Nikon, Inc., Melville, NY USA). A 12.5-mm core fiber-optic guided light (ThorLabs, Inc., Newton, NJ, USA) from the 150 W quartz halogen light bulb into the microscope condenser. This illuminator (DC-950H Dolan-Jenner DC- Regulated Fiber-Optic Illuminator, Edmund Optics, Inc., Barrington, NJ, USA) was operated at 90% of the light bulb’s maximum intensity. To control MSPRS positioning under incident excitation light, we custom-modified the stage of the microscope to increase its mechanical stability and to accommodate a T115 Nano-Stage (10 nm *xyz*-resolution) controlled by a Nano-Drive (Mad City Labs, Inc., Madison, WI, USA). Square (1/4 inch) rare-earth magnets fixed the MSPRS chip (Figure 1i) to the Nano-Stage. The microscope objective allowed us to focus on individual MSPRSs. The MSPRS emitted light from the microscope was fed through an optical fiber (0.34 NA, 600-μm core diameter) into a spectrometer (SpectraPro SP2150i, Teledyne Acton Optics, Acton, MA, USA) set with a diffraction grating (500 nm blaze-wavelength, 600 grooves/mm). The optical fiber axis was aligned to the optical axis of the microscope’s front port. The NA of optical fiber was larger than both objective’s NA and the spectrometer’s acceptance NA. To detect the MSPRS emitted light, we used an integrated photon-counting photomultiplier tube (PMT, PD-473, Teledyne Acton Optics, Acton, MA, USA) placed at the spectrometer’s exit slit set to 300 μm wide. Spectra Sense software (Teledyne Acton Optics, Acton, MA, USA) controlled the spectrometer, set the detector integration time, and recorded the spectra and the time series at fixed wavelengths.

The second setup (Figure 1j) used an inverted microscope (IMT2, Olympus Corporation of the Americas, Center Valley, PA, USA) with 0.3 NA condenser and an air objective (40×, 0.7 NA, Olympus Corporation of the Americas, Center Valley, PA, USA). We operated the microscope’s illuminator (50 W halogen light bulb) at 90% of the light bulb’s maximum intensity. To control MSPRS positioning under excitation light, we custom-modified the stage of the microscope to increase mechanical stability and to accommodate a mechanical XY translation stage with a 1-inch through-hole (ThorLabs, Inc., Newton, NJ, USA). The stage was driven by high-precision differential micrometer heads (0.5 μm resolution, ThorLabs, Inc., Newton, NJ, USA). Square (1/4 inch) rare-earth magnets fixed the MSPRS chip (Figure 1i) to the stage. A spectrometer (SpectraPro HRS 300, Teledyne Princeton Instruments, Trenton, NJ, USA) set with a diffraction grating (500 nm blaze-wavelength, 300 grooves/mm) was connected directly to the microscope to the left port through a C-mount. To detect the MSPRS emitted light, we used photon-counting avalanche photodiode (APD, SPCM-AQRH-15-FC, Perkin-Elmer Corp., Waltham, MA, USA) connected through an optical fiber to the spectrometer’s exit slit set at 300-μm width. Spectra Sense software controlled the spectrometer and set the detector integration time, record spectra, and time the series at fixed wavelengths.

Spectral data were collected at 2 nm increments from 475 nm to 700 nm with 1 s detector integration time with one exception. In that case, spectral data were collected at 5-nm increments from 480 nm to 680 nm at 1 s detector integration time to speed up spectra acquisition throughput. Spectral data were analyzed using Origin 6.1 (OriginLab Corp., Northampton, MA, USA). A 5-point adjacent averaging smoothing was applied to all data except the case mentioned before. All dimensional measurements were done in Fiji-ImageJ 1.52 (freeware, NIH, Bethesda, MD, USA). Each measurement reflects the mean and standard deviation over ten trials.

## 3. Conceptual Approach to MSPRS

The MSPRS’s complicated structure brings up several issues that need to be considered in order to further our understanding of MSPRS optical properties. Therefore, this section addresses several concepts that need to be brought into a MSPRS-specific context, and as such, not easily found in textbooks. The metal film thickness, its curvature, and its roughness are contributing factors that make the idealized Kretschmann model rather inaccurate. Furthermore, as presented below, surface roughness and metal film curvature are important mechanisms that excite SPPs at the metal surface, or couple SPPs back into propagating photons as being re-emitted rather than trivially transmitted through the film [32].

### 3.1. Surface Plasmon Resonance Overview

In metals, the conduction and valence bands overlap. The electron collective (the “sea of electrons”) can freely move in the bulk of a metal. Metals are modeled by Drude [33] as an electrically neutral plasma consisting of an electron gas at equilibrium surrounding positively charged metal ions that form the metal lattice. The smallest displacement of an electron, or a group of electrons, with respect to the metal lattice, results in a restoring Coulomb force set to bring the electron collective back to equilibrium. This disturbance sets the electron collective into a longitudinal oscillation that does not propagate; it is not described by a group velocity, a wave vector, nor a dispersion relation. The resonant frequency of such oscillation is called the plasma frequency [4]. Any other collective oscillation of the electron density at frequencies different from the plasma frequency is called a plasmon. Such oscillations are quantized in nature. The same way light is made of photon quanta, the charge density oscillation is made of plasmon quanta. Since the bulk plasmons are longitudinal oscillations, they cannot be excited or driven by incident electromagnetic waves that are transversal in nature. However, transverse magnetic (TM) electromagnetic waves can interact with plasmons at the bulk’s surface. The quanta of the surface charge density oscillations are called SPs. When SPs are excited by incident light in phase-matching mode (see Section 4.3.2), the frequency of the electromagnetic wave drives the frequency of the collective oscillation and the resonant coupling ‘surface plasmon – photon’ is called SPP or SPW [8]. Similar to evanescent waves, SPPs are described by wavelength, propagation length, and skin depth. By SPR, we imply that for both photon and SP the frequency and wave-vector component parallel to the interface are equal (also known as continuity equations at the interface). Both photon and SP have the same phase velocity but different group velocities. As such, the SPP is a surface-bound wave propagating at the metal interface and is described by a SPP wavelength [6]:

(1) $$\lambda_{SPP}=\lambda_{0}\sqrt{\frac{\varepsilon_{Au}^{Re}+\varepsilon_{d}}{\varepsilon_{Au}^{Re}\;\varepsilon_{d}}}$$

and a propagation length:

(2) LSPP=λ02π(εAuRe)2εAuIm(εAuRe+εdεAuRe εd)32

where $\lambda_{0}$ is the wavelength of the incident photon, $\varepsilon_{Au}^{Re}$ and $\varepsilon_{Au}^{Im}$ are the real and the imaginary parts of the complex relative permittivity of gold (Au) that is frequency-dependent, and $\varepsilon_{d}$ is the relative permittivity of the dielectric medium in contact with the metal surface. The SPP electric field reaches deep inside both materials at the interface, thus the SPP is also described by a penetration (skin) depth $\delta_{d}$ in dielectric and $\delta_{Au}$ in Au film [3]. Penetration depths are calculated as:

(3) $$\delta_{d}=\frac{\lambda_{0}}{2\pi \varepsilon_{d}}\sqrt{\varepsilon_{Au}^{Re}+\varepsilon_{d}}\;\mathrm{and}\;\delta_{Au}=\frac{\lambda_{0}}{2\pi \varepsilon_{Au}^{Re}}\sqrt{\varepsilon_{Au}^{Re}+\varepsilon_{d}}$$

### 3.2. Kretschmann Design

For most SPR introductory textbooks or review articles, KC has become the workhouse of this field [2,3,5,6]. KC became the platform for the first SPR-company, Pharmacia Biosensor AB, founded in 1984 to design, develop, and market the first SPR-biosensor instrument to measure real-time, label-free protein-protein interactions [34]. KC consists of a smooth flat thin metal film (Ag or Au coating), about 1 cm wide and 60–100 nm thick, deposited on a glass prism [34,35]. Two different interfaces are noted: the *incidence interface* at the boundary between prism and metal, and the SP *propagation interface* at the boundary between metal and a second dielectric medium with a refractive index less than the prism’s. In most biosensor applications, this medium is the analyte solution. The incidence interface is illuminated with p-polarized light through the prism at a precise angle of incidence, the *resonance angle* $\theta_{SPR}$. With these constraints in place, SPR is achieved and the energy of the incident photon is transferred to the SP producing a SPW.

**Figure 2:**
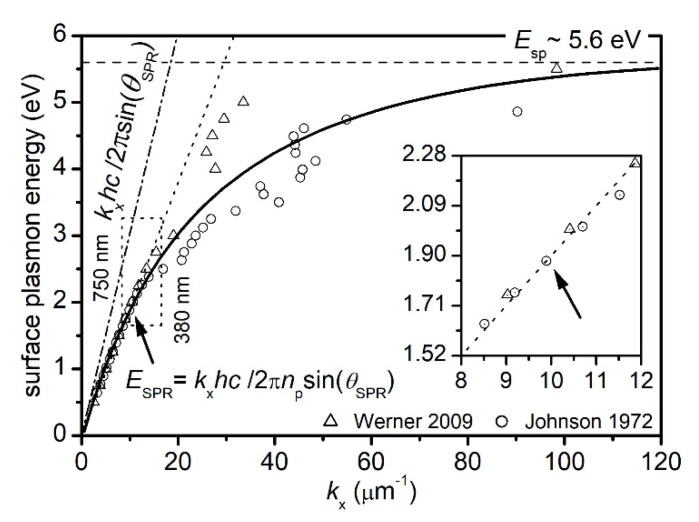
The SPR was designed based on the properties of the materials used in the fabrication of the MSPRS. The incident wavelength is of interest in studying the MSPRS resonance [27]: (**○**, **△**) the surface plasmons (SPs) dispersion relation in Au using experimental relative permittivity data from Johnson et al. [37], Werner et al. [38], and the calculations from Raether [6]; (**continuous line**) an idealized SP dispersion relation; (**dotted line**) the dispersion relation of p-polarized, 660-nm photons propagating through a polystyrene prism ($\varepsilon_{p}=2.55$) reaching resonance at $\theta_{\mathrm{SPR}}=40.58{}^{\circ}$; and (**dash-dotted line**) dispersion relation of the same photons at the same angle of incidence, but no prism ($\varepsilon_{p}=1.00$); (**dotted box**) the location of the visible spectrum, marked in wavelength units; (**long-dashed horizontal line**) the SP energy at large wave vectors. The arrows point at the SPR condition for the generation of a surface plasmon polariton (SPP) when the energy of the 660-nm photons travelling through a prism at the right angle of incidence ($\theta_{\mathrm{SPR}}$) excites a SP at the air/Au interface; (**insert**) Magnified view around the point of SP resonance.

However, this SPR canon builds a much-idealized model that comes in conflict with our understanding of more complex plasmonic structures such as the MSPRS presented in this paper [26,27,28,36]. For example, changes in the excitation method or the film thickness turn different results. In support of this statement, we introduce Figure 2 to compare the idealized SP dispersion relation (presented in many textbooks, Section 1) and two SP dispersion relations produced from experimental data [37,38,39,40]. These two sets of Au relative permittivity [37,38] were selected for comparison. While both sets are measured quantities, the experiments use highly different methods to excite SPs: photons vs. electrons. Johnson et al. use the normal-incidence reflection and transmission functions, whereas Werner at al. use reflection electron energy-loss spectroscopy. Optical constants of 40-nm Au films from Johnson et al. [37] and optical constants of 0.5-µm bulk Au from Werner et al. [38] were used to plot the dispersion relations of SPs using [6]:

(4) $$E_{SP}=\hbar \omega =\hbar ck_{x}^{Re}\sqrt{\frac{\varepsilon_{Au}^{Re}+\varepsilon_{d}}{\varepsilon_{Au}^{Re}\;\varepsilon_{d}}}$$

where $E_{SP}$ is the energy of the SPs, ℏ is the reduced Planck’s constant, ω is the SPP angular frequency, c is the speed of light in vacuum, $k_{x}^{Re}$ is the real part of the complex SPP wave vector propagating along *x*-axis parallel to the air/Au interface, $\varepsilon_{Au}^{Re}$ is the real part of the complex relative permittivity of Au that is frequency-dependent, and $\varepsilon_{d}$ is the relative permittivity of the dielectric sustaining SPP propagation.

SP energies are comparable at low wave numbers. At energies above 2.4 eV, the inter-band transitions introduce noticeable inconsistencies [41,42]. In Werner et al. [38], the energy of incident electrons is used to excite both SPs and longitudinal bulk plasmons, as well as in inter-band transitions to excite valence electrons from the 5sp to the 6d energy levels [41,42]. As a result, more energy is required before exciting a SPP of the same wave vector as in Reference [37]. At a higher wave vector, the SPPs become energy independent as they reach the SP oscillation frequency (at the air/Au interface) usually lower than the bulk plasmon excitations frequency. We followed Rakić et al. [43] as it models the dielectric function of Au while taking into account the inter-band transitions, and Raether [6] to evaluate the SP energy at large wave vectors:

(5) $$\hbar \omega_{SP}=\frac{\hbar \omega_{P}\sqrt{f_{0}}}{\sqrt{1+\varepsilon_{d}}}\;\sim \;5.6\;\mathrm{eV}$$

where the free-electron plasma energy is $\hbar \omega_{P}\;\sim \;9.03\;\mathrm{eV}$ ($k_{p}\;\sim \;45.8\;{\mu m}^{-1}$ or $\lambda_{p}\;\sim \;137.3\;\mathrm{nm}$), the intra-band transitions oscillator strength is $f_{0}\;\sim \;0.77$, and the air dielectric constant is $\varepsilon_{d}=$ 1.00.

Figure 2 shows that the non-radiative SP (continuous line) lying to the right of the photon dispersion relation (dash-dotted line) cannot be excited by incident photons in air. To excite a SP (Figure 2. dotted box) at resonance, the incident photon must gain momentum to match SP wave vector at the incidence interface. The slope of photon’s dispersion line must become less steep in order to intersect the SP dispersion curve (Figure 2. insert). A higher than air refractive index prism is needed to slow down the incident photons. When all constraints are met, the resonance (or the phase-matching) is accomplished and the energy of the incident photon is transferred to the excited SP generating the SPP [8]. According to the classic teachings, no photon should ever be reflected from this film [6]. However, this is only valid for smooth films, a consideration which is of great importance in instrument design [34]. Monitoring the drop in the intensity profile as a function of reflection angle allows for indirect monitoring of refractive index changes at the propagation interface.

### 3.3. Metal Thickness

The thickness of the metal film (infinitely wide, ~ 25 nm thick) affects the SPP properties. Several researchers show that as the film becomes thinner, the SPP degeneracy is lifted and two modes are now excited: a TM forward propagating, long-range, symmetric mode (as excited in the KC), and a TM back-propagating, fast attenuating, anti-symmetric mode [32,44,45,46]. As the width of the film becomes finite (about 1-µm) the two-dimensional confinement increases this degeneracy. Unlike their infinite width slab counterparts presented before, pure TM modes are not sustained by an ultra-thin metal film of finite width. The electric field has components both parallel and perpendicular to the metal surfaces, and all six field components are present for all modes. As the film thickness decreases below 80 nm, the coupling between the top and bottom surfaces gradually increases, and four modes become relevant to the SPPs propagation [32,45,47]. No photons are reflected nor refracted from these surfaces. There are always two leaky (radiative) wave solutions. For sufficiently thick films there are also two non-radiative waves. The symmetric and anti-symmetric modes excited in the MSPRS were previously addressed. It was concluded that in our geometry symmetric SP modes are preferentially excited [26].

### 3.4. Metal Surface Roughness

In the KC, the refractive index changes are revealed through monitoring the drop in reflected intensity as a function of incidence angle. In our geometry (see Section 4.2), as the flat surface turns into a spherical shell, monitoring the drop in reflected intensity is no longer possible. The main advantage of our setting was that the MSPRSs were operated in a transmission setting (see Section 2.4), a vertical sandwich with excitation source on top, MSPRS in the middle, and a detector at the bottom. Less noticed is the fact that Kretschmann and Raether [35] focus on studying the decay of excited SPP back into propagating photons. They show that the roughness of the surface decreases the SPP wave vector and, therefore, SPPs can re-emit into photons. As they adjust the incidence angle to reach $\theta_{SPR}$, they monitor the “transmitted” light on the other side of the thin flat film, and not the reflected light through the prism (as presented in most textbooks, Section 1). In a later work, Kretschmann [48] explains the surface roughness as a layer of vertical electrical dipoles that produce an additional surface current. In conclusion, both non-radiative and radiative SPPs are excited. Roughness is an important mechanism that scatters incident SPPs into other SPP modes or into emitted photons propagating away from the rough air-metal interface [35,48]. More recently, Passian et al. observe the interference pattern in the far-field radiation originating from roughness-induced scattering of SSPW in a Kretschmann setting by placing a microscope objective near the film surface. The far-field interference pattern yields a measure of the surface roughness [23].

**Figure 3:**
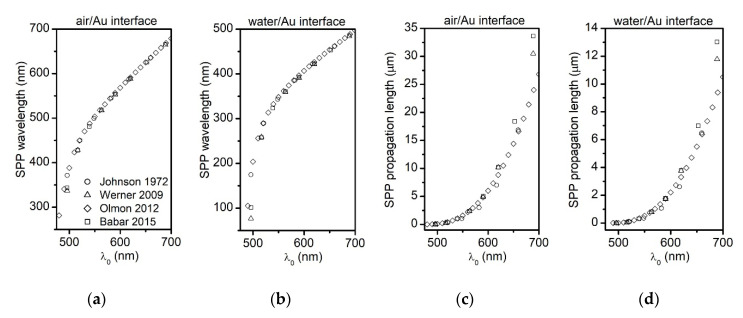
Four different sets of Au relative permittivity coefficients were used as a function of the incident photon wavelength: (**a**) the SPP wavelength at the air/Au interface; (**b**) the SPP wavelength at the water/Au interface; (**c**) the SPP propagation length at the air/Au interface; and (**d**) the SPP propagation length at the water/Au interface [37,38,39,40].

In practice, the biosensor fabrication method produces a sensor surface altered by uncontrolled coarseness or volume defects. The metal layer cannot always be annealed or recrystallized as higher temperatures could damage the biosensor. Aspnes et al. investigate the surface roughness and volume voids effects on Au’s relative permittivity (${\bar{\varepsilon }}_{Au}=\varepsilon_{Au}^{Re}+i\varepsilon_{Au}^{Im}$) and finds $\varepsilon_{Au}^{Re}$ to increase and $\varepsilon_{Au}^{Im}$ to decrease. In the inter-band region, such changes are independent of the void fraction [49]. Changes in the Au relative permittivity translate into changes in the SPPs properties. Surface roughness increases the number of intra-band collisions and the electron scattering processes at surface. These mechanisms dissipate the SPPs initial energy and as a result, the propagation length decreases [50]. As we later show, the surface of the MSPRS is rough and volume defects are visible. To investigate the extent of these contributions, we used complex relative permittivity values from four different sources [37,38,39,40] and plotted the SPP wavelength and the SPP propagation length in the visible spectrum from 475 nm to 700 nm at both air/Au interface (Figure 3a,c) and water/Au interface (Figure 3b,d). Since the data sets are in high agreement, our analyses and calculations below are based on the work of Olmon et al. because it offers a more discrete set of values [39].

### 3.5. Metal Surface Curvature

MSPRS has a curved propagation surface, it raises the concern whether SPP propagation along the meridians is sustainable. An over bent optical fiber cannot continuously maintain an evanescent field outside the fiber and the fiber leaks [51]. However, the SP dispersion relation presented by Raether shows no dependence on Cartesian coordinate axes [6]. Recently, Xiao et al. use cylindrical coordinates to investigate SSPWs on planar, U-, S-, G-, and L-curved graphene films with radii of 5, 50, and 500 nm (comparable in size with MSPRS). They find the dispersion relationships and propagation lengths for the different modes on cylindrical substrate are highly similar to those on planar substrates, and the bend of graphene nearly does not affect the dispersion relation even if the radius of substrate decreases to the nanometer scales [52]. Charbonneau et al. study the SPP propagation on both straight and curved waveguides [53]. They show in an S-curved waveguide, there is a transition loss at the meeting point of the two curved segments since curvatures have opposite directions. Berini and Lu employ cylindrical coordinates to study SPP propagation along 0.5 μm and 1.0 μm thick curved Au films [54] and conclude that while SPP propagation is sustainable, it is dominated by radiation loss rather than propagation attenuation for any curvature with a radius less than ~ 130 μm. For a curvature radius of 50 μm they find a transmission of about ItransmittedIincident ~ 0.15. Using a linear extrapolation of their findings down to 500 nm radius, we estimate a transmission of about $0.7\times 10^{-4}$, which is consistent with our earlier experimental findings [28]. The MSPRS light intensity with respect to incident light intensity is estimated at IMSPRSIincident=7.1×10−4 [28] (p. 17277). In conclusion, surface curvature sustains SSPW propagation, and along with surface roughness, is yet another mechanism that couples SPPs back into propagating photons.

## 4. Results and Discussion

### 4.1. MSPRS Design

In designing the MSPRS, we followed Kretschmann studies while aiming at ameliorating both geometrical and polarization constraints. The goal was to craft a smaller but sensitive biosensor. Measurements regarding the MSPRS sensing and biosensing capabilities, including its sensitivity to changes in the refractive index of medium and to biomolecular interactions at the sensor’s surface, were previously discussed [27,28]. As presented in Figure 2 (insert), the SP group velocity is only slightly different than the incident photon’s group velocity. Firstly, in practice, this translates to a drastic need for fine angular tunability. When studying small peptide interactions (molecular weight > 1000 Da), angular precision of 1 milli-degree is needed and it translates into a change in the medium’s refractive index of only $\Delta n=0.00001=10^{-5}\;RU$ (SPR response units) [55]. Secondly, in phase-matching mode or KC, SP can only be excited by p-polarized incident photons. Only one SPP mode can be excited at a time since any change in the photon wavelength is associated with a different $\theta_{SPR}$ angle.

**Figure 4:**
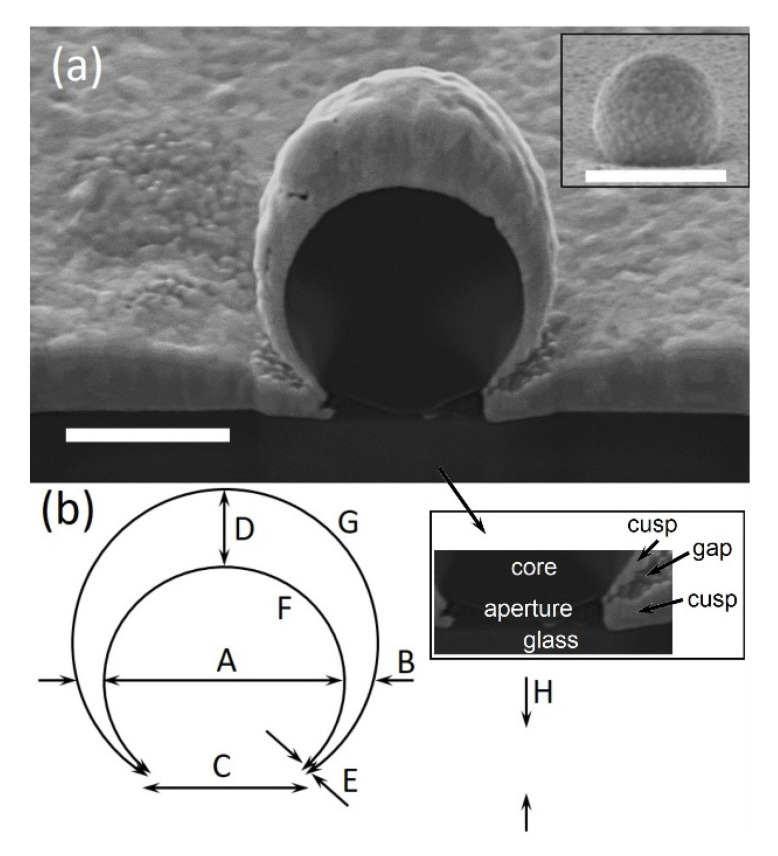
(**a**) Scanning electron micrograph of a vertical bisecting cross-section of a MSPRS made with a 780 ± 6 nm polystyrene sphere covered with a 170 nm Au film. Micrograph was corrected for 54° vertical tilt. Scale bar: 500 nm; (**top insert**) Scanning electron micrograph of a whole MSPRS, side view. Micrograph is tilt-corrected for 82° vertical tilt. Scale bar: 1 μm; (**b**) Template showing measurement locations; (**bottom insert**) Arrows point at aperture architecture details with labels.

The MSPRS spherical symmetry eliminates these constraints. Instead of a prism, one may consider a sub-micron sized spherical polystyrene sphere as a core, and instead of a thin flat Au film, consider a thin spherical shell wrapped around such core. Since the excitation photons must reach the incidence interface, which is now the *inner* surface of the Au shell, an underlying aperture is required at the bottom of the sphere to allow incoming photons to travel through the polystyrene core. By design, we end up with an *Omega configuration*, so-called because it resembles the Greek capital letter omega “Ω” in vertical cross-section (Figure 4a).

In this geometry, the angular constraint is lifted. As an example, let us consider the numerical parameters from Figure 2. When the entire sphere was illuminated vertically with 660-nm photons, SPR condition was met at multiple locations and for all the photons hitting the sphere on a circle of latitude described by a polar angle of θ=40.58°. In this work, we used a condenser lens illuminator with a NA=0.3. As such, the light illuminating the MSPRS comes within a vertical cone of light with an angle of $\varphi_{NA}=17.46{}^{\circ}$ with respect to the vertical axis in air. This makes the excitation domain degenerate from a circle of latitude into a spherical domain delimited by two circles of latitude described by polar angles of $\theta_{1}=23.12{}^{\circ}$ and $\theta_{2}=58.04{}^{\circ}$. When the MSPRS is inverted and illuminated through the glass substrate ($n_{g}=1.525$), the cone of light has a shallower angle $\varphi \prime_{NA}=11.35{}^{\circ}$ with respect to the vertical axis. This spherical domain is defined by two new circles of latitude described by the polar angles of $\theta_{1}=29.23{}^{\circ}$ and $\theta_{2}=51.93{}^{\circ}$. In this design, the excitation domain is independent of the diameter of the focusing spot of the incident light and only dependent of the NA of the illuminator that is usually more robust. Furthermore, this design allowed the use of non-polarized white light. All the photons hitting the excitation domain at $\theta_{SPR}$ have a component of the electric field vector that is p-polarized with respect to the point of incidence. While the maximum of the polarized to non-polarized light intensity ratio is 50%, the elimination of a polarizing filter simplifies the optical arrangement and enhances its robustness. Finally, the most significant gain in this design is the ability to use white light instead of monochromatic light. This is yet another advantage because multiple SP modes can now be excited on the same Au film and the need for fine geometrical alignment is eliminated. The excitation domains are overlapping. A calculation of the required incidence angles to achieve SPR revealed that for wavelengths ranging from 550 nm to 700 nm, the $\theta_{SPR}$ ranges from 43.19° to 40.63° [39].

### 4.2. MSPRS Anatomy

The concepts leading to MSPRS design seem straightforward, easy to implement, and offered major advantages. The experimental realization, however, presented a structure that was more complex than expected. Figure 4a is a scanning electron micrograph (SEM) of a dissected MSPRS. Following the template in Figure 4b, dimensional measurements of the MSPRS inner architecture were recorded (Table 1). Each dimension was measured ten times and the mean value and its standard deviation are reported below.

A measurement of the polystyrene core returned a diameter of 766±17 nm, which was in general accordance with the manufacturer’s specifications of 780±6 nm. As the Au is sputtered, it covered both the flat glass substrate and the polystyrene cores reaching deep under the core thus forming a subwavelength aperture. While the Au film deposition was experimentally set for a thickness of 170 nm on a flat surface, with ~ 3 nm of chromium underlayer, our measurements showed a consistent overall value of 180 ±7 nm. These two measurements were used as a control to validate the precision and accuracy of the rest of the measurements. The flat film within ~1 mm from the MSPRS was uniform in thickness, however, the film was dominated by roughness and volume defects, contributing to an increase in measurement error. Sputtering under an argon atmosphere is known to produce an outer surface dominated by 1–4 nm roughness [56]. Surrounding the MSPRS, roughly a core diameter from the symmetry axis, the flat film gradually wedges off to become much thinner under the core. The spherical shell was not uniform in thickness either. At the top, the Au film was thicker than the flat surrounding film reaching 204±17 nm. The shell narrows down to 68±2 nm around the core’s equator to finally reach a sharp cusp (Figure 4b-insert) deep under the core of E=20±6 nm thick. This curved shell was characterized by different parameters as measured between the cusps. First, the inner interface of F=1668±15 nm in length, and secondly, the outer interface of G=2241±43 nm in length, defined as the resonator sustaining SSPWs (Table 1). The underlying nano-aperture measured C=484±14 nm. However, other similar sensors revealed aperture diameters anywhere from 200 nm to 530 nm for similar core dimeters and Au film thicknesses. While the aperture was required by design, our study showed that the architecture of such aperture was very hard to control experimentally, and it was proved to be a source of major variations in MSPRS properties.

### 4.3. MSPRS Spectral Resonances–Classic Resonator

Mechanisms governing the formation of MSPRS resonances are not completely understood. A thorough understanding would imply our ability to predict and control MSPRS resonance’s mean wavelength, peak width, and peak intensity. MSPRS resonances have spectral line shapes resembling the Gaussian function. Similar to the normal distribution, the mean or expectation is represented here by the *mean wavelength* at which the spectral line reaches its maximum value called *peak intensity*. The spectral line full width at $e^{-0.5}$ of the full intensity is two times the standard deviation and it is called *peak width*. MSPRS resonances are governed by the interplay of several phenomena that make an analytical approach or simulation modeling rather difficult. Recent experimental evidence helped progress in understanding the MSPRS resonance properties. Each fabricated MSPRS substrate holds about 10,000 sensors and for each investigation, only one was selected. The fabricated MSPRSs were optically interrogated and the light emitted by individual MSPRSs was spectrally analyzed (see Section 2.4).

**Figure 5:**
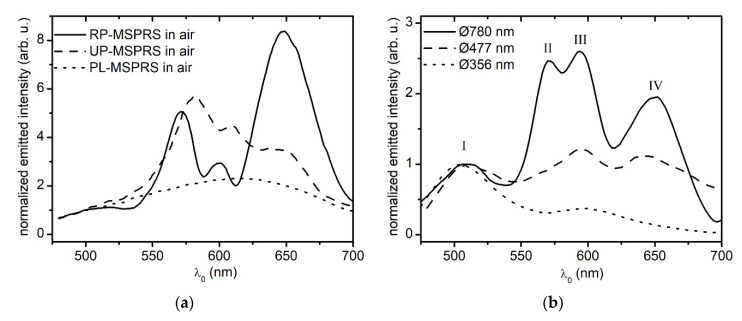
(**a**) MSPRS resonances displaying spectral lines with resolved peaks (**continuous line**), with un-resolved peaks (**dash line**) and peak-less (**dotted line**) profiles; (**b**) Spectral resonances of MSPRSs with different core diameters: (**continuous line**) three peaks for the 780-nm diameter; (**dash line**) two peaks for the 477-nm diameter, and (**dotted line**) one peak for the 356-nm core; For comparison, all spectra were normalized in intensity at 500 nm. To avoid clutter, only the continuous line spectrum in (**b**) had the peaks labeled with roman numbers (increasing from left to right), but the rule applied to all spectral profiles.

Figure 5a shows that the MSPRS emitted light is dominated by spectral peaks of somewhat arbitrary peak intensities. When visually inspected under the microscope, three main populations can be distinguished. As the background was much dimmer (dark green), our MSPRSs bear resemblance to stars against a clear night sky. The flat Au film had a transmission peak at ~ 500 nm, and it was about 100–500 times less intense than the MSPRS emitted light. One population appeared white and very dim, as compared to the others. When spectrally analyzed, these sensors showed no characteristic peaks, and their spectral profile remind us of the transmission spectrum through a neutral density filter. These peak-less MSPRSs (PL-MSPRSs) were not of interest and omitted in all other experiments (Figure 5a-dotted line). The second population appeared yellow-red and much brighter than PL-MSPRSs. When spectrally analyzed, these MSPRSs showed four un-resolved peaks (UP-MSPRSs) (Figure 5a-dashed line). The third population distinguished even more as they appeared more brownish and much brighter than both PL-MSPRSs and UP-MSPRSs. When spectrally analyzed, these MSPRSs showed four resolved peaks (RP-MSPRSs) (Figure 5a-continuous line). While UP-MSPRSs were omitted in our previous works [27,28], both UP-MSPRSs and RP-MSPRSs are of interest in the present study.

The excitation of standing mechanical waves on both elastic circular membranes [57] and shallow thin spherical shells [58,59,60,61] sparked our *hypothesis*: SSPWs were excited along the outer meridians of the Au shell between cusps. Although we are dealing with a different complexity, there are similarities between the MSPRS resonance properties and the vibration characteristics exited on elastic spherical shells. The oscillating electron collective can be treated as an elastic medium. Measurement G in Figure 4b was the resonator length. As such, MSPRS’s curved outer surface, delimited by the sharp cusps under the core, formed a “SPP curved cavity resonator”. The above hypothesis was tested several times through this work. For a better visual, one can refer to Xie et al. that shows the shape of axisymmetric modes of the vibrating spherical shells described by the frequency parameters $\Omega_{0m}$ [61] (p. 2321), Figure 5.

In a first approximation, one may consider a resonator in classic terms: a rectangular cavity resonator with highly reflecting walls perpendicular to the resonator’s length. If meridian G was that length, a SPP would propagate in one direction, reflect at the cusp, and propagate back while interfering constructively with itself to form a standing wave along G. Ideally, a SSPW mode would be excited when the optical path difference between incident wave and reflected wave was an integer number of SPP wavelengths:

(6) $$\delta \Lambda =2G=m\lambda_{SSPW}^{air}$$

where δΛ is the optical path difference inside the resonator, G is the resonator length in the direction of propagation between cusps, m is a positive integer and the harmonic’s index in air, and $\lambda_{SSPW}^{air}$ is the wavelength of a SSPW propagating at the air/Au interface G (Figure 4b). However, this classic rectangular cavity resonator model is highly idealized because, and as discussed in more detail later, the resonator length was always larger than the number of half SSPW wavelengths. Therefore, the concept of *effective resonator length* must be introduced:

(7) $$L=m\frac{\lambda_{SSPW}}{2}<G$$

With this rectangular cavity resonator model, two postulates were clear. Firstly, when the resonator length was continuously increased the wavelength of the excited harmonic mode was expected to proportionally increase. Secondly, by increasing the resonator length more harmonic modes would be excited inside the resonator.

#### 4.3.1. Core Diameter

While continuously increasing the core diameter would be very difficult to achieve experimentally because the cores were purchased (and only certain sizes were available), the second postulate was tested. Three different classes of MSPRSs were fabricated. From Figure 3a we learned that when excited with photons in the visible spectrum, the emitted MSPRS resonances corresponded to SSPWs of wavelengths ($\lambda_{SSPW}$) between 340 nm and 700 nm. Three core diameters were selected to allow the $\lambda_{SSPW}$ to fit inside the resonator G.

Figure 5b shows the spectra produced by MSPRSs with different resonator lengths. These spectra presented a different number of MSPRS resonances (spectral peaks) counted from left to right and indexed as I,II,III,etc. Each peak corresponded to an incident photon of wavelength $\lambda_{0}$ propagating in air that excited a SSPW of wavelength $\lambda_{SSPW}$ at the propagation interface G. Polystyrene nanoparticles with the 356±14 nm diameter cores were covered with 120 nm layer of Au, the 477±10 nm diameter cores were covered with 150 nm layer of Au, and the 780±6 nm diameter cores were covered with 170 nm layer of Au [31]. The Au film thickness had to be adjusted relative to core diameter. When the Au layer was too thin, transmission was stronger making the MSPRS resonances indistinguishable from background. Therefore, peak I (which was not a MSPRS resonance) dominated the entire spectrum and the MSPRS resonances were barely resolved (Figure 5b-dotted line). When the Au layer was too thick, MSPRS resonances were well resolved, but peak intensities were unnecessarily weakened due to film thickness attenuation (Figure 5b-continuous line).

Figure 5b-continuous line shows a spectrum with four peaks. The first peak at $\lambda_{0,I}\;\sim \;510\;\mathrm{nm}$ represented the light transmission through both flat film and curved shell. This peak is not Gaussian as it relates to the inter-band transitions [43]. As previously presented, this peak is not noticeably sensitive to changes in the refractive index at the propagation interface [27]. The second peak represented the first excited MSPRS resonance with a mean wavelength at $\lambda_{0,\mathrm{II}}\;\sim \;568\;\mathrm{nm}$. The third peak represented the second excited MSPRS resonance with a mean wavelength at $\lambda_{0,\mathrm{III}}\;\sim \;595\;\mathrm{nm}$. Furthermore, the fourth peak represented the third excited MSPRS resonance with a mean wavelength at $\lambda_{0,\mathrm{IV}}\;\sim \;648\;\mathrm{nm}$. These photons excited three different SSPWs at the air/Au interface with wavelengths of $\lambda_{SSPW,\mathrm{II}}\;\sim 528\;\mathrm{nm}$, $\lambda_{SSPW,\mathrm{III}}\;\sim 562\;\mathrm{nm}$, and $\lambda_{SSPW,\mathrm{IV}}\;\sim 623\;\mathrm{nm}$ (Figure 3b) [39]. While the core diameter was selected from a population of 780 nm core diameters, Figure 4 shows that the dissected MSPRS had a measured A=766 nm core diameter. The other two cores studied below required a geometrical correction. A geometrical model was built and validated for accuracy. The calculated resonator length was $G_{780}\;\sim \;2211\;\mathrm{nm}$ in agreement with the measured resonator length of $G_{766}=2241\pm 43\;\mathrm{nm}$. Such a large resonator allowed three MSPRS resonances to be excited. Firstly, we noted that as the core diameter decreased, the number of excited MSPRS resonances decreased (Table 2). From Equation (6) we inferred that the eighth harmonic ($m_{780,\mathrm{II}}=8$) of the $\lambda_{SSPW,\mathrm{II}}$, the seventh harmonic ($m_{780,\mathrm{III}}=7$) of the $\lambda_{SSPW,\mathrm{III}}$, and the sixth harmonic ($m_{780,\mathrm{IV}}=6$) of the $\lambda_{SSPW,\mathrm{IV}}$ were excited in the visible spectrum along the meridians of the 780 nm core resonator. Secondly, the resonator length $G_{780}$ was larger than all three effective resonator lengths: $L_{780,\mathrm{II}}\;\sim \;2112\;\mathrm{nm}$ for the first MSPRS resonance, $L_{780,\mathrm{III}}\;\sim \;1967\;\mathrm{nm}$ for the second MSPRS resonance, and $L_{780,\mathrm{IV}}\;\sim \;1869\;\mathrm{nm}$ for the third MSPRS resonance.

Figure 5b-dashed line shows a spectrum with three peaks. The first peak represents the Au film transmission. The second peak has a mean wavelength at about $\lambda_{0,\mathrm{II}}\;\sim \;595\;\mathrm{nm}$ and the third peak with a mean wavelength at $\lambda_{0,\mathrm{III}}\;\sim \;645\;\mathrm{nm}$. These photons would excite two different SSPWs at the air/Au interface with mean wavelengths of $\lambda_{SSPW,\mathrm{II}}\;\sim \;562\;\mathrm{nm}$ and $\lambda_{SSPW,\mathrm{III}}\;\sim \;620\;\mathrm{nm}$ (Figure 3b) [39]. Using the same procedure as before, we estimated the length of the resonator $G_{477}\;\sim \;2110\;\mathrm{nm}$ for the 477 nm core diameter. Another geometrical correction factor was considered because the Au film thickness represented 31.4% of the 477 nm core diameter, whereas, in Figure 3, the Au film thickness represented 23.5% of the 766 nm core diameter. When compared to Figure 3, the 477 nm core proved to be buried deeper in the flat film and had a shorter resonator length; however, it was larger than the 356 nm-core resonator length. As such, only two MSPRS resonance were excited. From Equation (6) we inferred that the seventh harmonic ($m_{477,\mathrm{II}}=7$) of the $\lambda_{SSPW,\mathrm{II}}$ and the sixth harmonic ($m_{477,\mathrm{III}}=6$) of the $\lambda_{SSPW,\mathrm{III}}$ were excited in the visible spectrum along the meridians of the 477 nm core resonator. Secondly, the resonator length $G_{477}$ was larger than both effective resonator lengths: $L_{477,\mathrm{II}}\;\sim \;1967\;\mathrm{nm}$ for the first MSPRS resonance and $L_{477,\mathrm{III}}\;\sim \;1860\;\mathrm{nm}$ for the second MSPRS resonance.

Figure 5b-dotted line shows a spectrum with two peaks produced by the shortest resonator length investigated in this work. The first peak again represents the Au film transmission and, therefore, not analyzed. The second peak has a mean wavelength at about $\lambda_{0,\mathrm{II}}\;\sim \;591\;\mathrm{nm}$. This photon excited one SSPW at the air/Au interface with a mean wavelength of $\lambda_{SSPW,\mathrm{II}}\;\sim \;558\;\mathrm{nm}$ (Figure 4a) [39]. Following the anatomy of MSPRS presented in Figure 4a, but rescaled to a 356 nm core diameter, we estimated the length of the resonator to $G_{356}\;\sim \;1588\;\mathrm{nm}$. A geometrical correction factor was considered because the Au film thickness represented 33.7% of the 356 nm core diameter, whereas, in Figure 4, the Au film thickness represented 23.5% of the 766 nm core diameter. Therefore, the 356 nm core was buried even deeper in the flat film and had a shorter resonator length. From Equation (6) we inferred that only the fifth harmonic ($m_{356,\mathrm{II}}=5$) was excited in the visible spectrum on the 356 nm core resonator. Secondly, the resonator length $G_{356}$ was larger than the effective resonator length: $L_{356,\mathrm{II}}\;\sim \;1395\;\mathrm{nm}$.

For the rest of the paper, and as previously discussed [27,28], our focus was to investigate the 780 nm core MSPRSs. When studied under visible light and for a narrow sub-micron range of core diameters, we noticed clear trends: an increase in the core diameter triggered an increase in the number of MSPRS resonances, from one to three. We learned that there was also an increase in the harmonic’s index, from 5 to 8. However, we were also presented with a new dilemma: the resonator length G was always larger than the effective resonator length L, as if there were other mechanisms at play “shortening” the length of the resonator. So far, we concluded that sustaining SSPWs along the Au shell meridians was feasible and SSPWs were responsible for the MSPRS resonances. The above experiment, while based on estimates and comparisons, was meant to show that our hypothesis had merit and it was worth perusing, thus motivating the next set of experiments. Table 2 above summarizes all the measurements and calculations presented in this section.

#### 4.3.2. Excitation Method Independence

**Figure 6:**
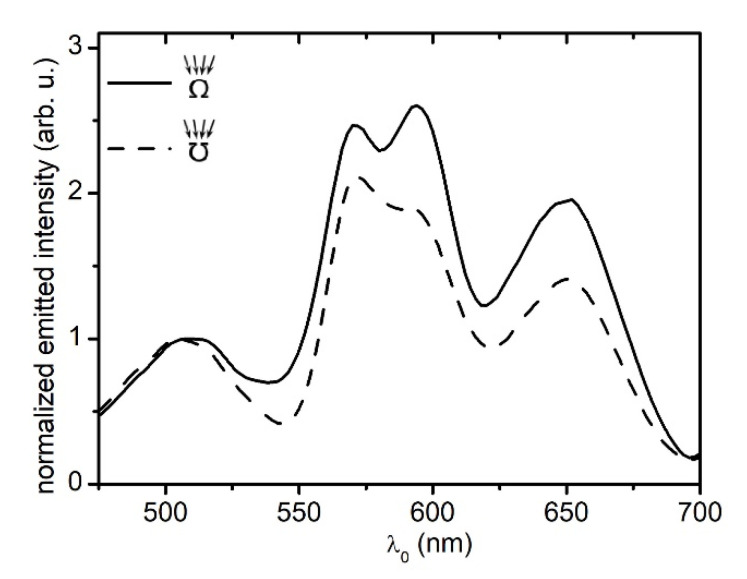
Spectral resonances of the resolved peaks (RP)-MSPRS from Figure 5b when excited by end-fire coupling configuration (continuous line) and Kretschmann-like configuration (dash line).

In a classical interference experiment where reflected or refracted waves constructively interfere with each other, such as thin-film interference or Newton’s rings setup, the point of incidence at the interface determines where the interference fringe is located. When the point of incidence changes location, the reflected and refractive waves propagate on different paths. While these new paths are parallel to the old ones, they start at a new location and, therefore, the fringe forms at new locations. Such experiments are dependent on excitation geometry. When a standing wave is excited inside a rectangular cavity resonator, the point of incidence is not affecting the standing wave formation. For example, the location a guitar string is pinched to generate a harmonic musical sound is never selected with the highest precision. Similarly, vibrations on circular membranes are independent of the excitation method. The same basic principle applies to SSPWs. To support our hypothesis, in Figure 6, we investigated MSPRS resonances generated by a 780 nm core diameter RP-MSPRS under two different excitation scenarios labeled as Ʊ-excitation and Ω-excitation. In a KC, the photon exciting SPP mode must travel through a prism before reaching the incidence interface. If the metal-prism configuration is flipped upside down, an incident photon in air cannot excite a SPP at the prism-metal interface (Figure 2, dash-dotted line).

KC is excitation-method dependent. Figure 6-dashed line shows that when the RP-MSPRS was illuminated through the underlying subwavelength aperture (Ʊ-excitation method), the KC conditions for exciting SPPs were met. The flat Au film was blocking the light and the only point of entry was through the underlying aperture. In our previous work, we mapped the light passing through a range of subwavelength apertures ranging from 110 nm to 770 nm [62]. We know for certain that exciting photons traveled through the core before reaching the incidence interface (see *F* in Figure 4b). A spectral four-peak Gaussian analysis $\left(R^{2}=0.997\right)$ revealed three MSPRS resonances with the following mean wavelengths (defined as normal distributions median) and peak widths (defined as two standard deviations): $\lambda_{0,\mathrm{II}}^{Ʊ}=568.2\pm 0.4\;\mathrm{nm}$ with $\Delta \lambda_{0,\mathrm{II}}^{Ʊ}=18.2\pm 0.6\;\mathrm{nm}$; $\lambda_{0,\mathrm{III}}^{Ʊ}=592.6\pm 0.7\;\mathrm{nm}$ with $\Delta \lambda_{0,\mathrm{III}}^{Ʊ}=31.2\pm 1.3\;\mathrm{nm}$; and $\lambda_{0,\mathrm{IV}}^{Ʊ}=649.0\pm 0.4\;\mathrm{nm}$ with $\Delta \lambda_{0,\mathrm{IV}}^{Ʊ}=42.6\pm 0.7\;\mathrm{nm}$.

However, when the RP-MSPRS was flipped (Ω-excitation method) and illuminated through air, photons were incident to the propagation interface instead (see *G* in Figure 4b). No SPPs should be excited as the Kretschmann SPR condition was not met. Figure 6-continuous line comes in contradiction with the classic SPR postulates because a MSPRS characteristic spectrum with four peaks was recorded as before. It seems that different excitation mechanisms were at play and our SSPWs were excited at the surface of the sensors in an end-fire coupling configuration (EFCC) [15,32]. Stegeman et al. [63] theoretically predicts 15 years after Kretschmann and Raether [35] that light focused in air at grazing incidence with respect to propagation interface excites symmetric SPPs modes in an EFCC. Such an excitation method is amenable to heterogeneous dielectric-metal-dielectric structures relying on spatial mode-matching rather than phase-matching. Yet another 17 years later, Charbonneau et al. come up with the first experimental observation of SPPs on thin flat Au films excited using an EFCC [64]. They show that as the incident photons travel farther and farther away from the propagation interface, the intensity of the SPPs diminishes. Charbonneau et al. use EFCC to more efficiently excite SPPs on thin curved Au films [53]. The recent work of Fisher et al. presents it the best: “*The end-fire method is simple and compact, making it an ideal coupling method for applications in which miniaturization is a priority*” [65] p.1044. EFCC does not require wave vector component matching between the incident photon and the SP, as prism, grating, or coupling via scattering off nanostructures do [65].

A four-peak Gaussian analysis $\left(R^{2}=0.997\right)$ revealed three MSPRS resonances with the following mean wavelengths and peak widths: $\lambda_{0,\mathrm{II}}^{\Omega }=568.4\pm 0.4\;\mathrm{nm}$ with $\Delta \lambda_{0,\mathrm{II}}^{\Omega }=20.3\pm 0.6\;\mathrm{nm}$, $\lambda_{0,\mathrm{III}}^{\Omega }=594.9\pm 0.7\;\mathrm{nm}$ with $\Delta \lambda_{0,\mathrm{III}}^{\Omega }=25.2\pm 0.7\;\mathrm{nm}$, and $\lambda_{0,\mathrm{IV}}^{\Omega }=648.0\pm 0.2\;\mathrm{nm}$ with $\Delta \lambda_{0,\mathrm{IV}}^{\Omega }=43.9\pm 0.5\;\mathrm{nm}$. When compared, the mean wavelengths and peak widths were in great agreement given the fact that spectral data was collected at 2-nm increments. Best fit analysis errors were less than the experimental errors. It can be concluded that the same SSPW modes were excited on the RP-MSPRSs independent of the excitation method. Figure 6 also shows that the peak intensity of the MSPRS resonances was more intense under EFCC excitation method, thus being consistent with Charbonneau et al. findings [53].

### 4.4. MSPRS Spectral Resonances–Reproducibility and Selectivity

**Figure 7:**
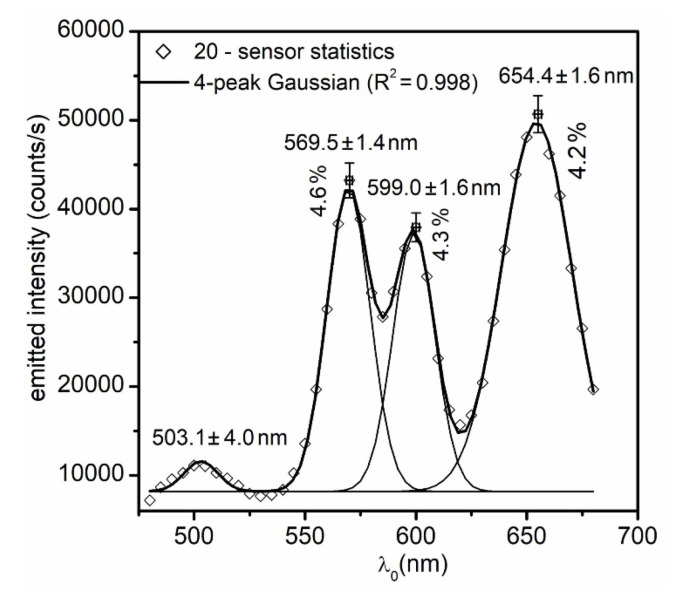
Reproducibility study over a population. (**◇**) experimental data comes from an average of 20 MSPRS spectra collected at 5 nm increments; (**thick continuous line**) A four-peak Gaussian statistical analysis of the experimental data; (**thin continuous lines**) Gaussian best fit of each MSPRS resonance in the experimental data; Error bars come from the four-peak Gaussian statistical analysis performed for each spectrum. Peak parameters were recorded and used to calculate experimental standard deviations in both wavelength and intensity.

To implement a new sensor technology and assure its acceptance in scientific research, reproducibility of the sensors, as well as the ability to select those with desired properties, is highly important. Reproducibility can be over the same batch (as each substrate holds about 10,000 MSPRSs) or across the batches (that would be highly important in industrial applications where a higher throughput and a production line must be maintained to strict specifications). This study focused on the first kind of reproducibility for several reasons. Several parameters drive the MSPRS optical properties: core diameter, film thickness, Au sputtering parameters (as they affect film roughness hence SPP emission efficiency), glass substrate preparation, and the intrinsic aperture architecture. Among all these parameters, the aperture architecture was the hardest to control, whereas the other parameters could be controlled with a higher precision. Once the aperture architecture is controlled for each sensor within a batch, the reproducibility across batches can be effectively tested. Therefore, our primary concern was: once a RP-MSPRS was selected, were we dealing with a unique sensor within the population, or did it belong to a sub-population of sensors all displaying highly similar properties? Secondly, how could we sort them out and how long would it take to find them among thousands in an academic laboratory setting? As a sub-population constraint, in this study, we were interested in selecting RP-MSPRSs that had the fourth peak well-defined (Figure 7).

We used the previously described *manual selection method* (see Section 4.3) and a new spectral method was specifically implemented in this study. Using the previous technique, about 500 MSPRSs were manually screened. Among them, 20 RP-MSPRSs were selected as they showed highly similar spectral properties. This manual method was very time consuming, tedious, and it was estimated to about 30 h of screening time. The *spectral method* allowed much faster screening and it is amendable for industrial settings. Since a representative RP-MSPRS showed a strong resonance of about 50,000 counts/s at 650 nm on a given substrate, the spectrometer’s dispersive grating was set at 650 nm and we ran it *live mode*. As we manually brought one sensor in focus at a time, we could rapidly screen for intensities of about 50,000 counts/s. Once the criteria were met, the RP-MSPRS spectrum was recorded and its position saved. Screening for 20 similar RP-MSPRSs took only one hour when we used the spectral method. Figure 7 shows the statistics over 20 selected RP-MSPRSs.

A four-peak Gaussian analysis $\left(R^{2}=0.998\right)$ was used to find the mean wavelength, the peak width, and the relative peak intensity of each RP-MSPRS in this set. These measurements represent the experimental outcomes. As such, the mean wavelengths of the MSPRS resonances were at $\lambda_{0,\mathrm{II}}^{air}=569.5\pm 1.4\;\mathrm{nm}$ with a relative intensity of ΔI0,III0,II=4.6%, also at $\lambda_{0,\mathrm{III}}^{air}=599.0\pm 1.6\;\mathrm{nm}$ with a relative intensity of ΔI0,III0,II=4.3%, and at $\lambda_{0,\mathrm{IV}}^{air}=654.4\pm 1.6\;\mathrm{nm}$ with a relative intensity of ΔI0,VII0,VI=4.2%. Although the aperture architecture is not directly controlled, it nonetheless seemed to produce sub-populations of RP-MSPRSs with highly similar properties. This study showed that MSPRSs resonance was reproducible, MSPRS selection was possible, and screening throughput could be dramatically improved.

### 4.5. MSPRS Spectral Resonances–Case Studies 

The rectangular cavity resonator model was implemented in Section 4.3. While the results support the hypothesis that SSPWs were sustained along the curved resonator G, we have also found that the resonator seemed universally larger than the effective resonator length L. This finding is not obvious, but it has merit. The rectangular cavity resonator model presented before did not account for the architecture of the underlying aperture and how it affected the resonator geometry. A closer look at Figure 4b-bottom insert shows that the curved Au film folds onto itself turning almost 360° at the cusps deep underneath the core. This created a metal-dielectric-metal (cusp-gap-cusp) configuration that brought uncertainty to the model. The cusp-gap-cusp composite was highly different from any vertical reflecting wall defining a rectangular cavity resonator. As such, the cusp-gap architecture and resulting optical properties are hardly controlled. Economou studies the dispersion relation for a metal-dielectric-metal configuration that resembles our cusp-gap-cusp architecture and finds that for thin dielectric layers with a thickness less than $\lambda_{p}$ ($k_{0}≲k_{p}$) and sandwiched between parallel metal films, all SPPs disappear because of the strong attenuation and enhanced retardation effects [44] pp. 547−549. Lu et al. show that intense near-fields are generated at circular sharp edge of the Au-cusps while studying highly similar structures (called nano-crescent moons) with a 300 nm dielectric core wrapped in a 100 nm Au film [66]. Since our sensor is standing on top of a flat conducting Au film, we do not know how these near fields are absorbed or reflected at the cusps, nor how deep the first and the last anti-nodes reach inside the gap as the standing wave forms along G [66]. Such complex mechanisms involved in “shortening” the length of the resonator affected the MSPRS resonance formation when the refractive index of the medium at the propagation interface changed. For a thorough investigation of such mechanisms, two *case studies* are presented below: a *case series* where the features of the case support this study’s hypothesis at MSPRS population level, and a *case report* where the study of the characteristics of a single MSPRS allowed a more in-depth analysis of the mechanisms driving the MSPRS resonance properties.

#### 4.5.1. MSPRS Resonances–Case Series 

In this section, we studied the MSPRS resonances in both air and water. We focused again on core diameters of 780 nm and Au layers of 170 nm and used the measurements presented in Figure 4 and Table 1 for guidance. Moreover, Figure 3 provided the SPP wavelengths and propagation lengths at the air/Au and water/Au interfaces. It was postulated that as the optical path increased, the SSPW wavelength decreased. Therefore, as G remained the same, a SSPW at the water/Au interface would be described by a shorter wavelength than a SSPW at the air/Au interface. Consequently, a SSPW mode excited in the water/Au resonator would be described by a larger harmonic’s index than the one of the SPPW mode excited in the air/Au resonator. Following Equation (6), the optical path difference equation is updated for two different media as follows:

(8) $$2G>2L=\delta \Lambda =m\lambda_{SSPW}^{air}=m\prime \lambda_{SSPW}^{water}$$

where L is the effective resonator length for each SSPW mode, m′ is the harmonic’s index in water, and $\lambda_{SSPW}^{water}$ is the wavelength of a SSPW propagating at the water/Au interface G (Figure 4b).

To enhance the accuracy of this study, a set of 25 RP-MSPRSs were selected out of a population of about 10,000 MSPRSs. This work followed the selection criteria developed in Section 4.4. For each selected RP-MSPRS, a spectrum in air was recorded and the sensor location was saved. Then, the microfluidic chip was flooded with water ($n_{w}^{25{}^{\circ}C}=1.333$) running at a rate of about 1 nL/s, thus changing the refractive index at the propagation interface. For exactly the same 25 RP-MSPRSs, a spectrum in water was then recorded. As presented in Figure 3b,d, the SSPWs at the water/Au interface had shorter wavelengths and propagation lengths than the SSPWs at the air/Au interface. Consequently, the photon wavelengths exciting MSPRS resonances at these two interfaces were shorter.

The four-peak Gaussian analysis performed for each of the 50 spectra allowed us to identify the mean wavelengths of each MSPRS resonance and their experimental standard deviations. The corresponding SSPW wavelengths were calculated after Olmon et al. [39] by using an adjacent-points linear fit of the data in Figure 3a for air and Figure 3b for water. To confirm the precision of this approach, we also generated a global 5th order polynomial regression best fit ($R^{2}=0.99994$) of the same experimental data. We found the first approach to be more precise and accurate then the polynomial regression analysis. The SSPW wavelengths uncertainty was estimated by performing two-dimensional error propagation calculations after Equation (1) with Au relative permittivity uncertainty $\delta \varepsilon_{Au}^{Re}=0.18$ [39] and the MSPRS resonance standard deviations determined from best-fit analysis.

In air, the first peak representing the transmission of the Au film was found at $\lambda_{0,I}^{air}=509.6\pm 2.8\;\mathrm{nm}$. The second peak representing the first excited MSPRS resonance had a mean wavelength at $\lambda_{0,\mathrm{II}}^{air}=573.2\pm 1.3\;\mathrm{nm}$. The third peak representing the second excited MSPRS resonance had a mean wavelength at $\lambda_{0,\mathrm{III}}^{air}=599.3\pm 2.2\;\mathrm{nm}$. The fourth peak representing the third excited MSPRS resonance had a mean wavelength at $\lambda_{0,\mathrm{IV}}^{air}=649.6\pm 2.3\;\mathrm{nm}$. These incident photons excited SSPWs at the air/Au interface with the mean wavelengths: $\lambda_{SSPW,\mathrm{II}}^{air}=535.3\pm 1.5\;\mathrm{nm}$, $\lambda_{SSPW,\mathrm{III}}^{air}=567.4\pm 2.2\;\mathrm{nm}$, and $\lambda_{SSPW,\mathrm{IV}}^{air}=624.6\pm 2.3\;\mathrm{nm}$.

In water, the first peak representing the transmission of the Au film was found at $\lambda_{0,I}^{water}=505.3\pm 0.9\;\mathrm{nm}$. The second peak representing the first excited MSPRS resonance had a mean wavelength at $\lambda_{0,\mathrm{II}}^{water}=588.9\pm 2.3\;\mathrm{nm}$. The third peak representing the second excited MSPRS resonance had a very low intensity and had practically disappeared. The four-peak Gaussian analysis could not be performed for all 25 spectra in water. Therefore, in order to remain consistent, a three-peak Gaussian analysis was run instead. The fourth peak representing the third excited MSPRS resonance had a mean wavelength at $\lambda_{0,\mathrm{IV}}^{water}=654.5\pm 1.7\;\mathrm{nm}$. These photons excited different SSPWs at the water/Au interface with wavelengths of $\lambda_{SSPW,\mathrm{II}}^{water}=395.3\pm 1.9\;\mathrm{nm}$, $\lambda_{SSPW,\mathrm{III}}^{water}=N/A$, and $\lambda_{SSPW,\mathrm{IV}}^{water}=457.8\pm 1.3\;\mathrm{nm}$.

From Figure 4 the measured resonator length $G_{766}=2241\pm 43\;\mathrm{nm}$, in agreement with the calculated resonator length $G_{780}=2211\;\mathrm{nm}$, was used as reference. The resonator sustained MSPRS resonances both in air and in water. From Equation (8) we inferred that for the first MSPRS resonance the eighth harmonic ($m_{780,\mathrm{II}}=8$) of $\lambda_{SSPW,\mathrm{II}}^{air}$ and the eleventh harmonic ($m\prime_{780,\mathrm{II}}=11$) of $\lambda_{SSPW,\mathrm{II}}^{water}$ were excited in the visible spectrum along the meridians of a 780 nm core resonator. The effective resonator length in air was $L_{\mathrm{II}}^{air}=2141\pm 12\;\mathrm{nm}$, whereas in water it was $L_{\mathrm{II}}^{water}=2174\pm 21\;\mathrm{nm}$ for the first MSPRS resonance. For the third MSPRS resonance, the sixth harmonic ($m_{780,\mathrm{IV}}=6$) of $\lambda_{SSPW,\mathrm{IV}}^{air}$ and the eighth harmonic ($m\prime_{780,\mathrm{IV}}=8$) of $\lambda_{SSPW,\mathrm{IV}}^{water}$ were excited in the visible spectrum along the meridians of a 780 nm core resonator. The effective resonator length in air was $L_{\mathrm{IV}}^{air}=1874\pm 14\;\mathrm{nm}$, whereas in water it was $L_{\mathrm{IV}}^{water}=1831\pm 11\;\mathrm{nm}$ for the third MSPRS resonance. This study is complementary to and supported our previously observed trends while extending the work to different media and improving on the precision and accuracy of our measurements. Table 3 summarizes all the measurements and calculations presented in this section.

The harmonic’s index is increasing, not only with the increase of the core diameter (see Section 4.3.1.) but also with the increase of the excitation wavelength. The same trend was now observed for different media: m increased from 8 to 11 in air and m′ increased from 6 to 8 in water. As the refractive index of the medium increased, the SSPW wavelength decreased, as predicted by Equation (8). In both media, the first MSPRS resonance, of a smaller wavelength, extended deeper inside the gap with $L_{\mathrm{II}}^{air}$ being only 4.5% shorter than G and $L_{\mathrm{II}}^{water}$ being only 3.0% shorter then G. On the other hand, the third MSPRS resonance, of a larger wavelength, did not reach as deep inside the gap with $L_{\mathrm{IV}}^{air}$ being 16.4% shorter than G and $L_{\mathrm{IV}}^{water}$ being 18.3% shorter then G. This discrepancy among SSPWs of different wavelengths made sense only when we considered that the gap between the Au-cusps is not uniform in thickness, but rather wedge-like and narrower as it reaches deeper under the dielectric core. Our conclusion that inside a metal-dielectric-metal configuration the phase velocity of the exciting photon was driven by the dielectric gap thickness was supported by Economou’s findings [44]. As the dielectric gap gets narrower and narrower, the SPP mode disappears. In conclusion, within the visible spectrum, inside our gap (a metal-dielectric-metal configuration) only the antisymmetric SPP mode is sustained whereas outside, between gaps (where there is a dielectric-metal-dielectric configuration) the symmetric SPP mode is sustained for an Au film thickness more than a λp2π. Therefore, the SPPs at these two interfaces are decoupled. As such, to excite a SPP of the same energy, a narrower gap requires a photon of a smaller wavelength, and vice-versa [44]. We proved that SSPWs excited along the Au shell meridians were holding for a population of MSPRSs. The SSPWs were responsible for the experimentally observed MSPRS resonances and their measured parameters were within the experimental uncertainties. These observed trends were steady across the population of MSPRSs with highly similar spectral properties as selected. These trends were also in agreement when different dielectric media were in contact with the sensors’ surface. The architecture of the gap between the Au-cusps explained the relationship between the effective resonator’s length L and the resonator’s length G, and thus could be used to predict MSPRS resonances of different wavelengths.

#### 4.5.2. MSPRS Resonances–Case Report

**Figure 8:**
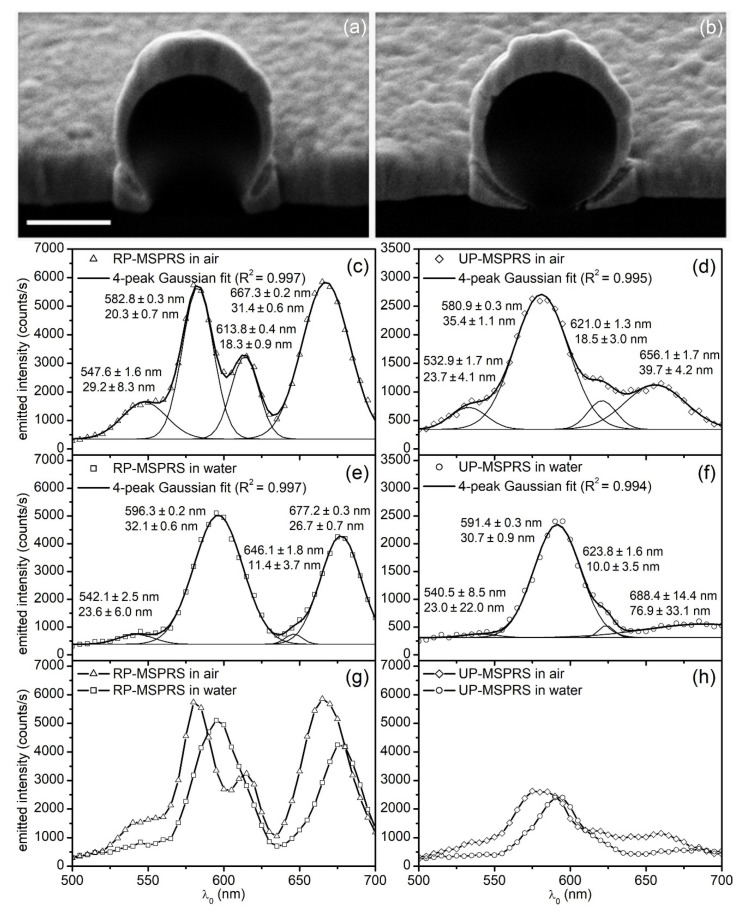
Scanning electron micrographs of vertical bisecting cross-sections of an RP-MSPRS (**a**) and an un-resolved peaks (UP)-MSPRS (**b**); Micrographs were corrected for 54° vertical tilt. Scale bar: 500 nm [36]; (**c**–**f**) MSPRS resonances (**symbols**), with associated Gaussian peak analysis (**thin lines**), and global best fit (**thick lines**) produced in air (**c**,**d**) and in water (**e**,**f**) by an RP-MSPRS (**c**,**e**) and UP-MSPRSs (**d**,**f**) are presented. Their mean wavelengths, peak widths, and standard deviations were included; (**g,h**) to facilitate visual comparison of the spectral and intensity shifts as the refractive index chanced, the MSPRS resonances from (**c,f**) were graphed at scale with interconnected data points.

The above findings, while consistent, were built around the dimensional analysis of the RP-MSPRS presented in Figure 4. In reality, minute variations in the underlying aperture architecture (Figure 8a,b) are responsible for generating sub-species of sensors, such as the UP-MSPRS and the RP-MSPRS. These differences must be investigated in depth for a better understanding of the MSPRS optical properties.

While Section 4.5.1 investigated how the underlying subwavelength aperture architecture drove such properties at population level, in this study we focused on how the aperture architecture drove the MSPRS optical properties at single-sensor level. For a thorough analysis, we selected two MSPRSs: one RP-MSPRS and one UP-MSPRS. These sensors were first interrogated in air, a spectrum was recorded, and their location saved. Then, the same sensors were interrogated in water and another spectrum was recorded. Finally, the same two sensors were dissected with the FIB and their anatomy studied. While following the nomenclature used in Figure 4b, the vertical cross-section of the RP-MSPRS (Figure 8a) and of the UP-MSPRS (Figure 8b) were examined and measurements of selected parameters were performed. MSPRS spectra were analyzed with a four-peak Gaussian fit to identify the mean wavelengths and associated peak widths (Figure 8c−f).

From Figure 8a we found that the RP-MSPRS had a dielectric core diameter of $A_{RP–MSPRS}=805\pm 29\;\mathrm{nm}$, an underlying aperture diameter of $C_{RP–MSPRS}=530\pm 7\;\mathrm{nm}$, a resonator length measured along the vertical meridian of $G_{RP–MSPRS}=2414\pm 29\;\mathrm{nm}$, a wedged gap with a length of $J_{RP–MSPRS}=211\pm 12\;\mathrm{nm}$, and a variable width from $E_{RP–MSPRS}^{min}≅22\;\mathrm{nm}$ at the deep end of the gap to $E_{RP–MSPRS}^{max}≅60\;\mathrm{nm}$ at the level of the flat Au-surface. The RP-MSPRS spectrum showed three MSPRS resonances in air with intensities of $I_{0,\mathrm{II},air}^{RP–MSPRS}≅5620\;\mathrm{counts}/s$, $I_{0,\mathrm{III},air}^{RP–MSPRS}≅3210\;\mathrm{counts}/s$, and $I_{0,\mathrm{IV},air}^{RP–MSPRS}≅5830\;\mathrm{counts}/s$ (Figure 8c) and three MSPRS resonances in water with intensities of $I_{0,\mathrm{II},water}^{RP–MSPRS}≅5020\;\mathrm{counts}/s$, $I_{0,\mathrm{III},water}^{RP–MSPRS}≅750\;\mathrm{counts}/s$, and $I_{0,\mathrm{IV},water}^{RP–MSPRS}≅4280\;\mathrm{counts}/s$ (Figure 7e).

From Figure 8b we found that the UP-MSPRS had a dielectric core diameter of $A_{UP–MSPRS}=800\pm 29\;\mathrm{nm}$, an underlying aperture diameter of $C_{UP–MSPRS}=204\pm 16\;\mathrm{nm}$, a resonator length measured along the vertical meridian of $G_{UP–MSPRS}=2895\pm 58\;\mathrm{nm}$, a wedged gap with a length of $J_{UP–MSPRS}=399\pm 30\;\mathrm{nm}$, and a variable width from $E_{UP–MSPRS}^{min}≅24\;\mathrm{nm}$ at the deep end of the gap to $E_{RP–MSPRS}^{max}≅64\;\mathrm{nm}$ at the level of the flat Au-surface. The UP-MSPRS spectrum showed three MSPRS resonances in air with intensities of $I_{0,\mathrm{II},air}^{UP–MSPRS}≅2710\;\mathrm{counts}/s$, $I_{0,\mathrm{III},air}^{UP–MSPRS}≅840\;\mathrm{counts}/s$, and $I_{0,\mathrm{IV},air}^{UP–MSPRS}≅1110\;\mathrm{counts}/s$ (Figure 8d) and three MSPRS resonances in water with intensities of $I_{0,\mathrm{II},water}^{UP–MSPRS}≅2340\;\mathrm{counts}/s$,$\;I_{0,\mathrm{III},water}^{UP–MSPRS}≅520\;\mathrm{counts}/s$, and $I_{0,\mathrm{IV},water}^{UP–MSPRS}≅550\;\mathrm{counts}/s$ (Figure 8f).

While the cores and Au-film properties selected in making these two sensors were statistically identical, the architectures of the underlying aperture were very different. Firstly, the aperture diameter was two to three times larger for the RP-MSPRS then for the UP-MSPRS, thus explaining why RP-MSPRS resonances were more intense than those of the UP-MSPRSs. This trend was first presented in Figure 5a, but is now further explored as the same MSPRS resonances were studied in both air and water. The aperture must be perceived as a “bottleneck” for either the exciting photons in the Ʊ-excitation configuration or the emitted photons in the Ω-excitation configuration (Figure 5c). Therefore, the peak intensities were affected. Comparing the peak intensities of the UP-MSPRS (Figure 8d) to those of the RP-MSPRS (Figure 8c) in air showed a $\frac{I_{0,\mathrm{II},air}^{RP–MSPRS}-I_{0,\mathrm{II},air}^{UP–MSPRS}}{I_{0,\mathrm{II},air}^{RP–MSPRS}}≅52\%$ decrease for the first MSPRS resonance, a $\frac{I_{0,\mathrm{III},air}^{RP–MSPRS}-I_{0,\mathrm{III},air}^{UP–MSPRS}}{I_{0,\mathrm{III},air}^{RP–MSPRS}}≅74\%$ decrease for the second MSPRS resonance, and a $\frac{I_{0,\mathrm{IV},air}^{RP–MSPRS}-I_{0,\mathrm{IV},air}^{UP–MSPRS}}{I_{0,\mathrm{IV},air}^{RP–MSPRS}}≅81\%$ decrease for the third MSPRS resonance. Comparing the peak intensities of the UP-MSPRS (Figure 8f) to those of the RP-MSPRS (Figure 8e) in water showed a $\frac{I_{0,\mathrm{II},water}^{RP–MSPRS}-I_{0,\mathrm{II},water}^{UP–MSPRS}}{I_{0,\mathrm{II},water}^{RP–MSPRS}}≅53\%$ decrease for the first MSPRS resonance and a $\frac{I_{0,\mathrm{IV},water}^{RP–MSPRS}-I_{0,\mathrm{IV},water}^{UP–MSPRS}}{I_{0,\mathrm{IV},water}^{RP–MSPRS}}≅87\%$ decrease for the third MSPRS resonance. Peak intensities of the second MSPRS resonance were very low (Figure 8e,f), making them impractical in any biosensor applications, therefore not discussed. While these particular percentages are not representative intrinsically, the trend they revealed was clear, highly representative, and observed constantly: as the mean wavelength of the SSPW got larger, the relative peak intensities got less intense. This trend became even clearer after the investigation of the gap configurations presented below.

Secondly, RP-MSPRS and UP-MSPRS had highly distinctive gap configurations. The RP-MSPRS revealed joint Au-cusps. As the Au-film reached underneath the core, it folded onto itself remaining continuous as the cusps join at the deep end. None of the UP-MSPRSs seemed to have joint Au-cusps. The Au-film seemed to reach much deeper underneath the core with a gap length almost twice as long as the RP-MSPRS gap. The UP-MSPRS Au-cusps were much sharper and slender, thus increasing the difficulty of measurement and resulting in larger standard deviations. While the gaps’ minima and maxima widths were comparable, the UP-MSPRS increased gap length made this wedge less steep. We estimated wedge angles of $\alpha_{RP–MSPRS}≅10.1{}^{\circ}$ and $\alpha_{UP–MSPRS}≅5.7{}^{\circ}$. This evidence supported the hypothesis presented in Section 4.5.1 that the cusp-gap-cusp configuration made the SPP mode disappear forcing the symmetric SPP mode to form mostly outside the gap where the dielectric-metal-dielectric configuration existed. While the size of these configurations was about the same ($G_{RP–MSPRS}-2J_{RP–MSPRS}=1992\pm 43\;\mathrm{nm}$ vs. $G_{UP–MSPRS}-2J_{RP–MSPRS}=2097\pm 118\;\mathrm{nm}$), and responsible for the number of MSPRS resonances and their mean wavelengths, the wedged cusp-gap-cusp configurations at the end of the resonator G were, therefore, responsible for the resolvability of the MSPRS resonances in a spectrum. This can be better understood if we used a simple analogy: Consider only one harmonic standing wave excited in a rectangular cavity resonator (Section 4.3). Such wave would have a very well-defined mean wavelength and no peak width (i.e., delta function). However, when the vertical walls of the resonator lean inwards, turning the rectangle into a trapezoid, standing waves of the same harmonic’s index, but slightly different wavelengths, can now be excited inside the resonator. Since these SSPWs are excited by incoherent incident photon, no interference is expected. The spectral resonance that resembled the delta-function turned into a Gaussian function described by a lower peak intensity and a non-zero peak width. The spectral line broadened because the first and the last nodes of the standing wave form at slightly different locations on the tilted walls. The same way, the wedged cusp-gap-cusp configurations affect the formation of the SSPWs along the resonator G and, therefore, contributed to the broadening of the MSPRS resonances. Since the UP-MSPRS have more shallow gaps, their MSPRS resonances experienced more broadening. Figure 8c,f presents the peak width for every MSPRS resonance for both UP-MSPRS and RP-MSPRS, and one can see that UP-MSPRS has relatively wider peaks. This effect was amplified as the wavelength increased, as explained by Economou [44]. A shorter wavelength would “reach” deeper inside the wedged gap, while the shallower gap angle, combined with the roughness of the Au-cusps, increased the uncertainty of mean wavelength location. MSPRS resonance broadening was also observed in a peak separation study.

To investigate the spectral peak separation (in wavelength), we used concepts from spectroscopy and chromatography. We qualitatively described the ability to resolve two Gaussian spectral lines by defining the peak resolution as:

(9) $$R=\frac{\left|\lambda_{1}-\lambda_{2}\right|}{2\sigma_{1}+2\sigma_{2}}=\frac{\left|\lambda_{1}-\lambda_{2}\right|}{\Delta \lambda_{1}+\Delta \lambda_{2}}$$

where $\lambda_{1}$ and $\lambda_{2}$ are the mean wavelengths of adjacent peaks, $\Delta \lambda_{1}$ and $\Delta \lambda_{2}$ are the peak widths at $e^{-0.5}$ of the maximum peak intensity. We considered two adjacent peaks to be well resolved when R≥1, however in practice, we were able to differentiate peaks with less resolution. Peak resolutions for adjacent MSPRS resonances emitted by RP-MSPRS and UP-MSPRS, in both air and water, are summarized in Table 4.

The same trend was observed: RP-MSPRS (with shorter and more abrupt gaps) had better resolved resonances (Figure 8a,c,e). While the first MSPRS resonance was relatively well separated for both sensors in both media, the third MSPRS resonance was distinguishable for RP-MSPRS only. For both sensors, the second MSPRS resonance had an almost undetectable peak in water (Figure 7e,f). It is important to notice that the first and third MSPRS resonances would be good choices in biosensor applications. In our prior work, only the third MSPRS resonance was used for real-time monitoring of biochemical interactions [27,28,36].

Thirdly, following the same steps as in Section 4.5.1, we measured the resonator G for both RP-MSPRS and UP-MSPRS and evaluated the effective resonator lengths L in air and in water. Our findings are summarized in Table 5.

The RP-MSPRS showed a consistent decrease in harmonic index and effective resonator length L as the mean wavelength increased. This trend was observed for all three MSPRS resonances, in both media. As we investigated each MSPRS resonance with respect to the refractive index increase, we noticed that while the mean wavelength $\lambda_{0}$ red-shifted (Figure 8g) and the harmonic index m increased by 2–3 increments, the effective resonator lengths were similar to one another for this RP-MSPRS: $\frac{L_{0,\mathrm{II},air}^{RP–MSPRS}-L_{0,\mathrm{II},water}^{RP–MSPRS}}{L_{0,\mathrm{II},air}^{RP–MSPRS}}≅1.2\%$, $\frac{L_{0,\mathrm{III},air}^{RP–MSPRS}-L_{0,\mathrm{III},water}^{RP–MSPRS}}{L_{0,\mathrm{III},air}^{RP–MSPRS}}≅0.9\%$, and $\frac{L_{0,\mathrm{IV},air}^{RP–MSPRS}-L_{0,\mathrm{IV},water}^{RP–MSPRS}}{L_{0,\mathrm{IV},air}^{RP–MSPRS}}≅0\%$.

As the SSPWs formed along the resonator G, the effective resonator length either matches the wedge-free segment of the resonator (G−2J) or it penetrates inside the wedges. As such, the first RP-MSPRS resonance reached about 5% inside the gaps in air and about 5.6% inside the gaps in water. The second RP-MSPRS resonance reached about 1.3% inside the gaps in air and about 0.9% inside the gaps in water. From gap prospective, the first RP-MSPRS resonance penetrated about 47% of the $J_{RP–MSPRS}$ in air and about 53% of the $J_{RP–MSPRS}$ in water, whereas the second MSPRS penetrated about 13% of the $J_{RP–MSPRS}$ in air and about 8% of the $J_{RP–MSPRS}$ in water. However, the third RP-MSPRS resonance did not reach inside the gaps being about 3% shorter than the G−2J length. We can only hypothesize that such setting was responsible for the large variations in the third resonance peak intensity revealed from investigations of Figure 5a-continuous line, Figure 7, and Figure 8c as compared to their first MSPRS resonance peak intensities. For its ability to produce highly intense and well-resolved peaks, the third MSPRS resonance was preferred in our past biosensor applications [27,28,36].

Instead, the UP-MSPRS showed inconsistent decrease in harmonic index and the effective resonator length L as the mean wavelength increased. This deviation from the above-observed trend was responsible for the lower intensity in the second MSPRS resonance, in both media. Once again, we noticed that while the mean wavelength $\lambda_{0}$ red-shifted (Figure 8h) and the harmonic index m′ increased, the effective resonator lengths were similar to one another for this UP-MSPRS: $\frac{L_{0,\mathrm{II},air}^{UP–MSPRS}-L_{0,\mathrm{II},water}^{UP–MSPRS}}{L_{0,\mathrm{II},air}^{UP–MSPRS}}≅0.4\%$, $\frac{L_{0,\mathrm{III},air}^{UP–MSPRS}-L_{0,\mathrm{III},water}^{UP–MSPRS}}{L_{0,\mathrm{III},air}^{UP–MSPRS}}≅0.3\%$, and $\frac{L_{0,\mathrm{IV},air}^{UP–MSPRS}-L_{0,\mathrm{IV},water}^{UP–MSPRS}}{L_{0,\mathrm{IV},air}^{UP–MSPRS}}≅0.8\%$.

As before, we observed that the first UP-MSPRS resonance reached about 4% inside the gaps in air and about 4.3% inside the gaps in water, very similar to the first RP-MSPRS resonance behavior. However, the second UP-MSPRS resonance reached about 13% inside the gaps in air and about 12.7% inside the gaps in water, which is dissimilar to the second RP-MSPRS resonance behavior. The third UP-MSPRS resonance reached about 5.4% inside the gaps in air and about 4.6% inside the gaps in water, again dissimilar to the third RP-MSPRS resonance behavior that formed outside the gaps. From gap prospective, first UP-MSPRS resonance penetrated about 10% of the $J_{UP–MSPRS}$ in air and about 12% of the $J_{UP–MSPRS}$ in water, the second UP-MSPRS penetrated about 34% of the $J_{UP–MSPRS}$ in air and about 33% of the $J_{UP–MSPRS}$ in water, whereas the third MSPRS penetrated the gaps about 14% of the $J_{UP–MSPRS}$ in air and about 12% of the $J_{UP–MSPRS}$ in water. The trend observed before, that as the mean wavelength increases, the SSPWs ability to penetrate the wedged gap decreases, was not applicable for the UP-MSPRS resonances [44]. The experimental findings were evident: the second and the third UP-MSPRS resonances had less intense and broader peaks. It became obvious that the resonant conditions prescribed by Equation (9) were not met, thus these SSPWs were poorly excited in UP-MSPRSs. This study was particularly important because it revealed that the first UP-MSPRS resonance had similar trends to the first RP-MSPRS resonance and, therefore, it could reliably be used in future biosensor application. By using both RP-MSPRS and UP-MSPRS, we double biosensor production throughput.

## 5. Conclusions

MSPRS were developed to improve the sensitivity, while miniaturizing the sensor footprint, of the classic real-time, label-free surface plasmon resonance sensors. The journey from the drawing board to experimental sensor was short, but the practical realization presented us with a more complex scenario than anticipated. A thorough understanding would imply our ability to ultimately predict and control MSPRS resonance mean wavelength, peak width, and peak intensity. In this work, we discussed the mechanisms governing the MSPRS resonances, and a range of experiments was designed and implemented to allow us to understand the MSPRS spectra. As we transitioned from the KC towards the MSPRS, we found that Au film thickness, curvature, and roughness were contributing factors that made the classic model rather inaccurate. An extensive literature exploration was necessary for understanding how such factors supported our main hypothesis: the SSPWs are responsible for the formation of the MSPRS resonances that are sensitive to changes in refractive index at sensor’s surface. Our work showed that an increase in the core diameter triggered an increase in the number of excited MSPRS resonances. The SSPW modes were independent on the excitation method and the EFCC excitation method produced more intense MSPRS resonances. We found the resonator length to be always larger than the number of half-wavelengths formed along the resonator. While controlling the aperture architecture remains a challenge, we proved that sub-populations of MSPRSs with highly reproducible properties could be identified and used reliably. The morphology of the dielectric gap sandwiched between the Au-cusps proved to be responsible for the MSPRS peak broadening and intensity. When comparing the RP-MSPRS and UP-MSPRS (since they present different aperture architectures) we showed that as the mean wavelength of the SSPW got larger, the relative peak intensities got less intense. Consequently, RP-MSPRS with shorter and more abrupt gaps revealed more resolved MSPRS resonances. While the first MSPRS resonance was relatively well separated for both RP-SMPRS and UP-MSPRS in both media, only the third MSPRS resonance was distinguishable for RP-MSPRS. For both types of sensors, the second MSPRS resonance had an undetectable peak in water. This study revealed that both the first and third MSPRS resonances would be good choices for biosensor applications since in our prior work, only the third MSPRS resonance was used for real-time monitoring of biochemical interactions. We conclude that sustaining SSPWs along the Au shell meridians is feasible and the decoupling of the SSPWs back into propagating photons is responsible for the MSPRS resonances and their characteristic spectra, while the architecture of the sub-wavelength underlying aperture, both its diameter as well as the shape of the cusp-gap-cusp configuration, drastically affect the mean wavelength, peak width, and peak intensity describing the MSPRS resonances.

## Figures and Tables

**Table 1:** Dimensional measurements of parameters in Figure 4 that define the MSPRS architecture.

Parameters (nm)	A	B	C	D	E	F	G	H
mean	766	902	484	204	20	1668	2241	180
SD ^1^	17	6	14	17	6	15	43	7

^1^ SD stands for *standard deviation*.

**Table 2:** Summary of concepts, numerical estimates, and measurements discussed in Section 4.3.1.

**Core Size (nm)**	356 ± 14	477 ± 10		780 ± 6		
**Peak Index**	*II*	*II*	*III*	*II*	*III*	*IV*
**Resonance Index**	1st	1st	2nd	1st	2nd	3rd
$\lambda_{0}$ **(nm)**	~591	~595	~645	~568	~595	~648
$\lambda_{SSPW}$ **(nm)**	~588	~562	~620	~528	~562	~623
***M***	5	7	6	8	7	6
***L* (nm)**	~1395	~1967	~1860	~2112	~1967	~1869
***G* (nm)**	1588 ± 30	2110 ± 40		2241 ± 43		

**Table 3:** Summary of concepts, numerical calculations, and measurements discussed in Section 4.5.1.

**Core Size (nm)**	780 ± 6			
**Interface**	air/Au		water/Au	
**Peak Index**	*II*	*IV*	*II*	*IV*
**Resonance Index**	1st	3rd	1st	3rd
$\lambda_{0}$ **(nm)**	573.2 ± 1.3	649.6 ± 2.3	588.9 ± 2.3	654.5 ± 1.7
$\lambda_{SSPW}$ **(nm)**	535.3 ± 1.5	624.6 ± 2.3	395.3 + 1.9	457.8 ± 1.3
**Harmonic Index**	8	11	6	8
***L* (nm)**	2141 ± 12	1874 ± 14	2174 ± 21	1831 ± 11
***G* (nm)**	2241 ± 43			

**Table 4:** Resolution of adjacent MSPRS resonances for RP-MSPRS and UP-MSPRS in air and water.

*II vs. III*	*III vs. IV*
$R_{\mathrm{III}–\mathrm{II},air}^{RP–MSPRS}=0.803$	$R_{\mathrm{IV}–\mathrm{III},air}^{RP–MSPRS}=1.076$
$R_{\mathrm{III}–\mathrm{II},air}^{UP–MSPRS}=0.744$	$R_{\mathrm{IV}–\mathrm{III},air}^{UP–MSPRS}=0.603$
$R_{\mathrm{III}–\mathrm{II},water}^{RP–MSPRS}=1.145$	$R_{\mathrm{IV}–\mathrm{III},water}^{RP–MSPRS}=0.816$
$R_{\mathrm{III}–\mathrm{II},water}^{UP–MSPRS}=0.786$	$R_{\mathrm{IV}–\mathrm{III},water}^{UP–MSPRS}=0.743$

**Table 5:** Summary of concepts, calculations, and measurements for both sensors studied in Section 4.5.2.

Sensor Type	RP-MSPRS						UP-MSPRS					
***C* (nm)**	805 ± 29						800 ± 29					
***J* (nm)**	211 ± 12						399 ± 30					
**Interface**	air/Au			water/Au			air/Au			water/Au		
**Peak Index**	*II*	*III*	*IV*	*II*	*III*	*IV*	*II*	*III*	*IV*	*II*	*III*	*IV*
**Resonance Index**	1st	2nd	3rd	1st	2nd	3rd	1st	2nd	3rd	1st	2nd	3rd
$\lambda_{0}$ **(nm)**	582.8 ± 0.3	613.8 ± 0.4	667.3 ± 0.2	596.3 ± 0.2	646.1 ± 1.8	677.2 ± 0.3	590.9 ± 0.3	621.0 ± 1.3	656.1 ± 1.7	591.4 ± 0.3	623.8 ± 1.6	688.4 ± 14
$\lambda_{SSPW}$ **(nm)**	547.3 ± 0.4	584.3 ± 0.4	644.0 ± 0.2	403.0 ± 0.3	450.3 ± 1.9	477.6 ± 0.3	545.0 ± 0.4	592.6 ± 1.4	631.7 ± 1.6	398.0 ± 0.4	429.9 ± 1.6	487.1 ± 13
**Harmonic Index**	8	7	6	11	9	8	8	8	7	11	11	9
***L* (nm)**	2190 ± 3	2045 ± 3	1932 ± 1	2217 ± 3	2026 ± 17	1932 ± 3	2180 ± 3	2370 ± 11	2211 ± 11	2189 ± 3	2364 ± 18	2192 ± 117
***G − 2J* (nm)**	1992 ± 53						2097 ± 118					
***G* (nm)**	2414 ± 29						2895 ± 58					
